# Upregulation of the Ca_v_1.3 channel in inner hair cells by interleukin 6‐dependent inflammaging contributes to age‐related hearing loss

**DOI:** 10.1111/acel.14305

**Published:** 2024-08-15

**Authors:** Mingshun Lu, Fuyu Xian, Xishuo Jin, Guodong Hong, Xiaolong Fu, Shengnan Wang, Xinyu Li, Haichao Yang, Hongchen Li, Haiwei Zhang, Yuxin Yang, Jundan Xiao, Hui Dong, Yaling Liu, Haitao Shen, Ping Lv

**Affiliations:** ^1^ Department of Pharmacology, The Key Laboratory of Neural and Vascular Biology, Ministry of Education, The Key Laboratory of New Drug Pharmacology and Toxicology, The Hebei Collaboration Innovation Center for Mechanism, Diagnosis and Treatment of Neurological and Psychiatric Disease Hebei Medical University Shijiazhuang Hebei China; ^2^ Medical Science and Technology Innovation Center Shandong First Medical University & Shandong Academy of Medical Sciences Jinan Shandong China; ^3^ Shandong Provincial Hospital, Medical Science and Technology Innovation Center, College of Clinical and Basic Medicine Shandong First Medical University & Shandong Academy of Medical Sciences Jinan Shandong China; ^4^ Department of Neurology, Aerospace Center Hospital, School of Life Science Beijing Institute of Technology Beijing China; ^5^ Department of Neurology, The Second Hospital of Hebei Medical University, The Key Laboratory of Neurology, Ministry of Education Hebei Medical University, Neurological Laboratory of Hebei Province Shijiazhuang Hebei China; ^6^ Lab of Pathology Hebei Medical University, Shijiazhuang Hebei China; ^7^ Hebei Collaborative Innovation Center of Tumor Microecological Metabolism Regulation Affiliated Hospital of Hebei University Baoding Hebei China

**Keywords:** age‐related hearing loss, Ca_v_1.3 channel, hair cells, interleukin 6

## Abstract

Age‐related hearing loss (AHL) is the most common sensory disorder amongst the older population. Inflammaging is a ≈chronic low‐grade inflammation that worsens with age and is an early sign of AHL; however, the underlying mechanisms remain unclear. We used electrophysiological and genetic approaches to establish the importance of interleukin 6 (IL‐6)‐dependent inflammation in AHL. Elevated IL‐6 in the cochlea enhanced Ca_v_1.3 calcium channel function in the inner hair cell (IHC) synapse in mice with AHL. IL‐6 upregulated the Ca_v_1.3 channel via the Janus kinase‐mitogen activated kinase pathway, causing neurotransmitter excitotoxicity and synapse impairment; IL‐6 deficiency or the administration of a Ca_v_1.3 channel blocker attenuated this age‐related damage, and rescued hearing loss. Thus, IL‐6‐dependent inflammaging upregulated the Ca_v_1.3 channel in IHCs, contributing to AHL. Our findings could help the comprehensive understanding of inflammaging's effects on AHL, aiding in early intervention to protect against hearing decline.

## Graphical abstract

The age‐related hearing loss mice displayed inflammatory infiltration in the cochlea associated with elevation of the IL‐6 and enhancement of the Ca_v_1.3 calcium channel in inner hair cells. IL‐6 up‐regulation of Ca_v_1.3 channels via the JAK‐MAPK pathway contribute to neurotransmitter excitotoxicity and synaptic impairment in the cochlea of mice with deafness. IL‐6 deficiency or administration of a Ca_v_1.3 channel blocker attenuated this damage and rescued hearing loss.

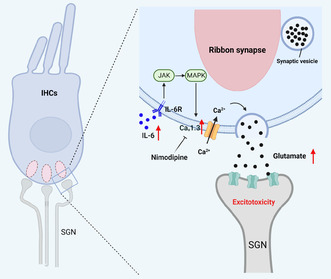

**Abbreviations**
ABR: auditory brainstem response
AHL: age‐related hearing loss
HL: hearing loss
IH: improved hearing
IHC: inner hair cell
IL‐6: interleukin 6
JAK: Janus kinase
KO: knockout
MAPK: mitogen‐activated kinase
MBP: myelin basic protein
NF‐κB: activated the nuclear factor kappa B
SGN: spiral ganglion neuron
TNF‐α: tumor necrosis factor α

## 1 INTRODUCTION

Age‐related hearing loss (AHL), known as presbycusis, is a progressive, irreversible, and symmetrical bilateral sensorineural hearing loss. It is the most common sensory disorder in older adults (Collaborators, 2021). The World Health Organization has reported that approximately one‐third of people over 65 years are affected by disabling hearing loss. Untreated AHL contributes to social isolation and depression, increasing the long‐term risk of cognitive decline, and dementia (Livingston et al., 2020; Rutherford et al., 2018). AHL involves numerous risk factors ranging from genetics to environmental stressors; however, the underlying mechanisms remain unclear.

Inflammaging has been characterized in various age‐related diseases, such as cardiovascular disease, type II diabetes, and Alzheimer's disease (Blasko et al., 2004; Grant & Dixit, 2013; Osiecki, 2004). Inflammaging is defined as a chronic low‐grade inflammation that worsens with age (Baylis et al., 2013; Franceschi et al., 2018). It arises from immunosenescence or cellular senescence of the immune system and contributes to the pathogenesis of various age‐related diseases (Baylis et al., 2013; Franceschi et al., 2018). Increasing evidence has shown that inflammaging is also linked to AHL (Seicol et al., 2022). For example, an increased enrichment of genes associated with immune responses and inflammatory pathways has been observed in the cochleae of AHL mice (Su et al., 2020). Macrophage numbers and morphology indicate age‐related changes in the lateral wall and auditory nerve of human cochlear tissues (Noble et al., 2019). Tumor necrosis factor α (TNF‐α), interleukin 6 (IL‐6), and IL‐1β were upregulated in the aging cochleae (Lyu et al., 2020; Verschuur et al., 2012), supporting the notion that inflammaging may be an underlying mechanism for AHL. However, Wu et al. reported that low concentrations of TNF‐α activated the nuclear factor kappa B (NF‐κB) transcription, which mediates hair cell survival instead of injury (Wu et al., 2022), indicating that low‐grade inflammation may play a profound role in AHL. Moreover, macrophage activation precedes sensory cell pathogenesis (Frye et al., 2017), suggesting that inflammaging is an early sign of AHL. Nevertheless, how inflammaging contributes to hearing impairment in AHL remains to be investigated.

In the cochlea, time and intensity of sounds are encoded at ribbon synapses formed by the inner hair cells (IHCs) and spiral ganglion neurons (SGNs). Specifically, sound‐induced displacement of the stereociliary bundles leads to IHC depolarization and the activation of Ca_v_1.3 calcium channels clustered at the cell's presynaptic active zones (Jeng, Ceriani, Hendry, et al., 2020; Platzer et al., 2000). Consequently, Ca^2+^ influx into the IHCs via Ca_v_1.3 channels controls the glutamate release from synaptic vesicles. Subsequently, postsynaptic SGNs receive input from IHCs via ribbon synapses and relay the auditory information to the brainstem. Notably, dysfunction of IHC ribbon synapses occurs earlier than IHCs or auditory nerve deficits (Sergeyenko et al., 2013; Xiong et al., 2020), making an early event in AHL. Therefore, this study aimed to explore the roles of inflammaging in AHL ribbon synapse impairment. Our findings could reveal novel mechanisms underlying early hearing impairment in AHL, which may provide new therapeutic opportunities for targeted interventions to limit inflammation.

## 2 MATERIALS AND METHODS

### 2.1 Animals

All experimental animal protocols were approved by the Animal Care and Ethics Committee of Hebei Medical University (Shijiazhuang, China) (2022148). C57BL/6 mice (C57) were obtained from the Charles River Laboratories. IL‐6 knockout (IL‐6 KO) mice were obtained from the Institute of Laboratory Animal Sciences of the Chinese Academy of Sciences. All mice were bred under a 12 h: 12 h light–dark cycle.

### 2.2 Auditory brainstem response testing

Auditory brainstem response (ABR) measurements were performed as previously described (Shen et al., 2018). Briefly, mice were anesthetized with ketamine (100 mg/kg) and xylazine (10 mg/kg) via intraperitoneal injection. Platinum needle electrodes were inserted subcutaneously at the vertex of the head (reference), ipsilateral mastoid (recording), and contralateral hind legs of the mice (ground). ABR was measured using a TDT system (Tucker‐Davis Technologies, Gainsville, FL, USA). The lowest intensity required to generate a reproducible ABR waveform was defined as the hearing threshold. Responses were measured for clicks and pure tones at various frequencies. Latencies of ABR wave I were measured at a 90 dB click. The amplitudes were calculated as the peak‐to‐peak amplitude of the next negative trough.

### 2.3 Quantitative real‐time polymerase chain reaction

Total RNA samples from mouse cochlea (four to five mice were used for each sample) were extracted using an RNAeasy Micro Kit (Qiagen, Hilden, Germany). Total RNA was reverse transcribed using PrimeScript reverse transcriptase (#RR036Q; TaKaRa). Quantitative polymerase chain reaction (PCR) was performed using a TB GREEN Kit (Takara Bio Inc., China) with a two‐step cycling program and a Bio‐Rad CFX Connect Real‐Time PCR system. Relative gene expression was analyzed and calculated with glyceraldehyde 3‐phosphate dehydrogenase (*GAPDH*) as the housekeeping gene using the formula: 2^−ΔΔCt^. All primers used in this study were designed using Primer Premier 6.25 (PREMIER Biosoft International, CA, USA) and synthesized by Sangon Biotech (Shanghai, China). The following primer sequences were shown in Table S1.

### 2.4 Immunofluorescence

The cochleae were fixed in 4% paraformaldehyde and decalcified in 10% EDTA at 4°C. Then, the specimens were processed using 10% and 30% sucrose gradients and embedded in an optical cutting temperature compound (Tissue‐Tek) for cryosectioning in the modiolar plane. The specimens were incubated with primary antibodies overnight at 4°C. The sections were rinsed with PBS three times and incubated with secondary antibodies for 1.5 h at room temperature. Nuclei were counterstained with 4,6‐diamidino‐2‐phenylindole dihydrochloride (DAPI; #D9542; Sigma) for 10 min at room temperature. Images were analyzed using a Leica LAS AF Lite and processed using ImageJ and Photoshop CS5. The number of synaptic ribbons puncta (CtBP2^+^, GluR2^+^) was measured using the 3D‐Objects Counter Plugin for Images. The antibodies were list in Table S2.

### 2.5 SGN morphometry and counting

To evaluate SGN morphometry and density, cochlear sections were stained with hematoxylin and eosin (H&E). The Rosenthal canal was divided into three regions: the apex, middle, and base. SGN density in these three regions was measured using Image‐Pro Plus 5.1. Cells in one field (apex, middle, or base) were counted in each section, and six representative sections were analyzed in one cochlea per mouse.

### 2.6 Western blot

Cochlear tissues (five mice per sample) were homogenized, and cells were lysed in a RIPA solution containing 0.1% SDS, 50 mM Tris (pH 7.4), 1 mM EDTA (pH 8.0), 1% sodium deoxycholate, 1% TritonX‐100, and 200 μM of phenylmethanesulfonyl fluoride (PMSF). The lysis mixture was centrifuged at 13,800 × *g* for 30 min at 4°C, and protein concentration in the supernatant was determined using a BCA protein assay kit (#23235, Thermo, CA). Equal amounts of proteins were resolved by SDS‐PAGE and transferred onto a PVDF membrane. The membranes were blocked with 5% dry milk in TBST for 2 h at room temperature and then rinsed three times with TBST. Then, the membranes were probed with anti‐IL‐6 (#66146‐1‐lg, Proteintech, 1:200) overnight at 4°C. A fluorescent secondary antibody (IRDye800CW, LI‐COR, Biosciences, NE) was applied for 90 min at room temperature. Western blotting was performed using Image Lab 4.0.

### 2.7 Isolation of SGNs

SGNs were isolated from the cochlea of mice following a detailed procedure outlined in a previous study (Lv et al., 2010). Briefly, mice were humanely sacrificed, and the temporal bones were removed. The SGN tissue were digested in an enzyme mixture containing collagenase type I (1 mg/mL) and DNase (1 mg/mL) at 37°C for 15 min. After gentle trituration and centrifugation (2000 rpm for 5 min) in 0.45 M sucrose, the cell pellets were reconstituted in 900 μL of culture media (Neurobasal™ A, supplemented with 2% B27 (v/v), and 0.5 mM L‐glutamine; Invitrogen). SGNs were kept in culture for 24–48 h prior to electrophysiological recordings to allow for the detachment of Schwann cells from the soma.

### 2.8 Electrophysiology

For Ca^2+^ current recordings, the specimens were continuously perfused with an extracellular solution containing (mM): 105 NaCl, 35 TEA‐Cl, 2.8 KCl, 10 CaCl_2_, 1 MgCl_2_, 10 HEPES, and 10 D‐glucose. Amino acids and vitamins were added to the extracellular solution. The pH was adjusted to 7.3. The pipette solution contained (mM): 140 Cs‐gluconate, 10 TEA‐Cl, 10 HEPES, 1 MgCl_2_, 2 MgATP, and 0.3 NaGTP, adjusted to pH 7.2 with CsOH. IHCs were held at −86 mV with step voltages ranging from −86 mV to +34 mV, with 10‐mV increments. Recordings were performed at room temperature using a Multiclamp 700 B amplifier (Molecular Devices, Sunnyvale, CA). Series resistance and capacitance compensation (>70%) were performed, and signals were filtered at 2 kHz using a low‐pass Bessel filter and digitized at a rate of ≥20 kHz using a Digidata 1440A (Axon Instruments) digitizer and pClamp 10.7 software (Molecular Devices, Sunnyvale, CA).

Capacitance (Cm) measurements of the IHCs were performed using the same pipette solution and the extracellular solution used for the Ca^2+^ current recordings. The EPC‐9 amplifier (HEKA Elektronik, Lambrecht, Germany) was controlled by the “Pulse” software (HEKA). The currents were sampled at frequencies of 20–40 kHz and low‐pass filtered at frequencies of 2–5 kHz. Cm was determined using the Lindau–Neher approach (Lindau & Neher, 1988), which was implemented in the “Pulse” software‐locking module. A 1 kHz, 70 mV peak‐to‐peak sinusoid was applied around a DC holding potential of −80 mV.

P21‐30 WT C57 mice were used for the IL‐6 incubation experiments. Ca^2+^ currents were recorded after incubation with IL‐6 (100 ng/mL) (GenScript, Z03189‐25, NJ, USA) for 30, 60, and 90 min. The following compounds were bath‐applied: IL‐6, 10 μM AG490 (MCE, HY‐12000, NJ, USA), or 10 μM PD98059 (MCE, HY‐12028, NJ, USA), or 20 μM Stattic (MCE, HY‐13818, NJ, USA). The effect of IL‐6 on synaptic vesicle release was evaluated by recording changes in the membrane capacitance of IHCs.

To record Ca^2+^ currents, cultured SGNs were held at −50 mV and stepped to depolarizing voltage steps from −50 mV to 30 mV, using a ΔV of 10 mV. The pipette solution contained the following (mM): 60 CsCl, 75 N‐methyl‐D‐glucamine (NMDG), 3 MgCl_2_, 10 HEPES, 10 EGTA, and 5 ATP‐Mg, adjusted to pH 7.2 with CsOH. The extracellular solution contained (mM): 120 choline chloride, 20 TEA‐Cl, 2 CaCl_2_, 10 HEPES, and 5 D‐glucose, adjusted to pH 7.4.

### 2.9 In vivo drug administration

Seven‐month‐old C57 mice were divided into four groups: WT 9 M, solvent, 5 mg/kg nimodipine, and 10 mg/kg nimodipine. Nimodipine was obtained from MCE (HY‐B0265, NJ, USA), dissolved in a solvent (5% Tween‐80, 10% DMSO, 40% PEG 300, and 45% saline), and administered intraperitoneally every other day for 60 days. Solvent mice were injected intraperitoneally with the same volume of solvent. ABR was recorded after treatment with nimodipine to assess hearing function. The cochlea was removed on Day 60 to evaluate morphological changes in the SGNs and functional changes in the ribbon synapses.

### 2.10 Statistical analysis

All data were presented as the mean ± SEM. Statistical analyses were performed using GraphPad Prism (version 8.0.2.; GraphPad Software, Inc., San Diego, CA, USA). Student's *t* test was used to compare data between the two groups, and *ANOVA* was used for multiple comparisons. The Kruskal–Wallis test was used for comparisons of data with non‐normal distributions or heterogeneity of variance. *, **, and *** indicated statistically significant results compared to appropriate controls and indicated *p* < 0.05, *p* < 0.01, and *p* < 0.001, respectively.

## 3 RESULTS

### 3.1 Increased inflammatory infiltration with elevated IL‐6 in the cochlea of AHL mice

**FIGURE 1:**
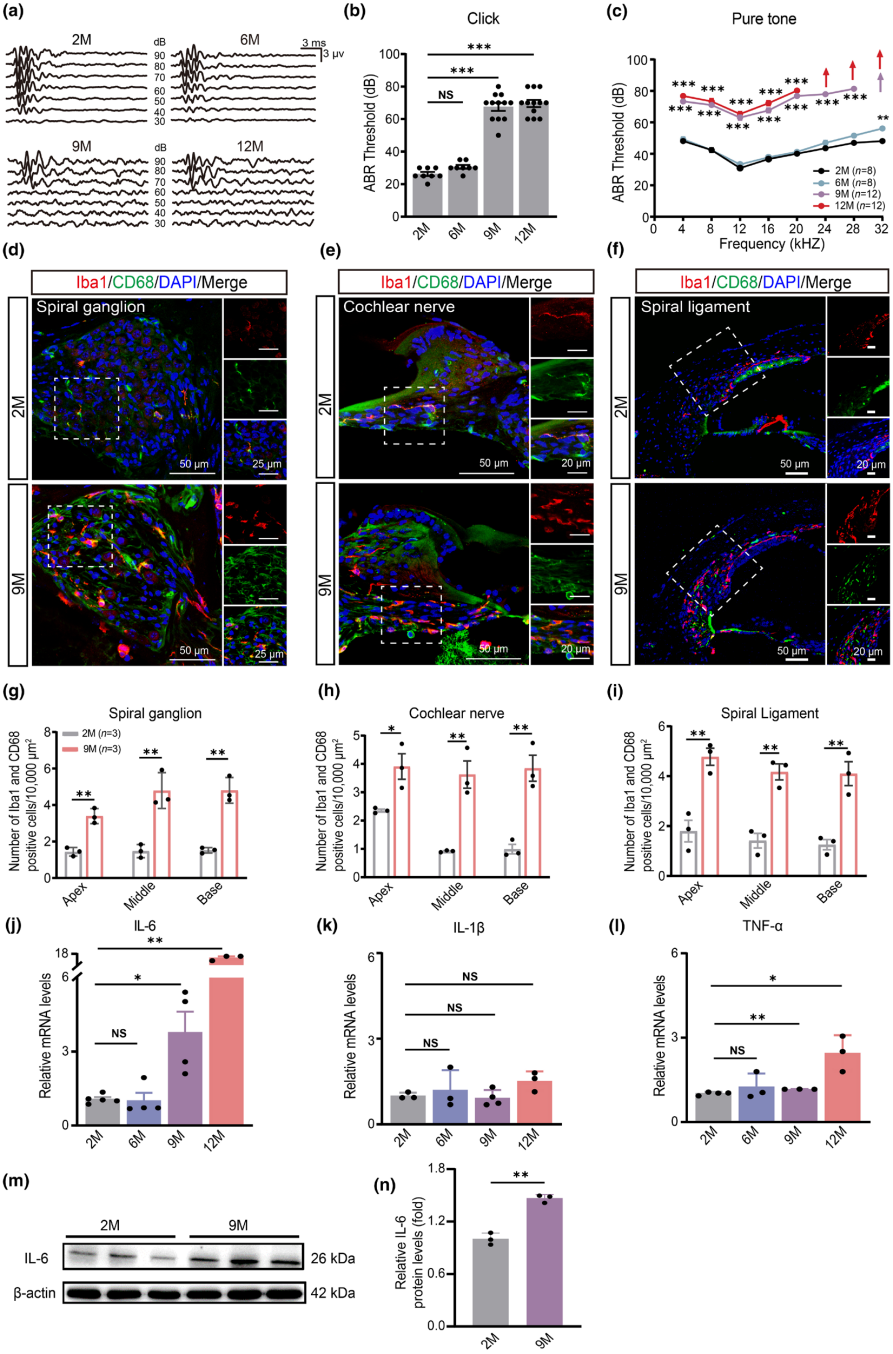
Increased inflammatory infiltration with elevated IL‐6 in the cochlea of AHL mice. (a) Representative ABR waveforms respond to click stimuli in 2‐, 6‐, 9‐ and 12‐month‐old WT mice. (b, c) Click values and ABR thresholds were measured in WT mice at 2–12 months old. (d–f) Confocal images showing expression of Iba1(red) and CD68 (green) in the spiral ganglion, cochlear nerve and spiral ligament of 2‐ and 9‐month‐old WT mice. (g–i) Iba1/CD68‐ double positive cells are quantified in the apical, middle, and basal cochlea of 2‐ and 9‐month‐old WT mice (*n* = 3). (j–l) Expression of IL‐6, IL‐1β, and TNF‐α in cochlea in 2‐, 6‐, 9‐ and 12‐month‐old WT mice as determined by real‐time PCR. (m, n) Expression of IL‐6 in protein levels in cochlea of 2‐ and 9‐month‐old WT mice (*n* = 3) as determined by Western blotting. Data are means ± SEM, *p < 0.05, **p < 0.01, ***p < 0.001.

To explore the role of inflammaging in AHL, we first evaluated the hearing function in C57 mice of varying ages by measuring ABR (Figure 1a–c). Compared with 2‐month‐old mice, 9‐month‐old mice displayed significantly elevated ABR thresholds across all frequencies in response to click (Figure 1b) and pure‐tone stimuli (Figure 1c), indicating hearing impairment.

We examined inflammatory infiltration in the cochlea of mice with AHL using immunofluorescence. The number of macrophages (CD68 and Iba1‐positive cells) was quantified in the spiral ganglion, cochlear nerve, and spiral ligament at the apical, middle, and basal turns of the cochlea, respectively (Figure 1d–i). As shown in Figure 1d,g a significant increase was observed in macrophage infiltration in the spiral ganglion region of 9‐month‐old mice compared to 2‐month‐old mice. Similarly, we observed a significant increase in the number of macrophages in the cochlear nerve (Figure 1e,h) and spiral ligament (Figure 1f, i) of 9‐month‐old mice, indicating the presence of inflammation in the cochlea.

To further understand the role of inflammatory cytokines in AHL‐related cochlear inflammation, we used real‐time PCR to assess the expression of three key cytokines, IL‐6, IL‐1β, and TNF‐α, in the cochlea. Cochlear IL‐6 levels were higher in mice at 9 months of age (Figure 1j), correlating with elevated hearing thresholds. TNF‐α levels were enhanced in 12‐month‐old mice, while IL‐1β levels did not change in mice aged 2–12 months (Figure 1k,l). Furthermore, increased IL‐6 expression in the cochlea of 9‐month‐old mice was confirmed by western blotting (Figure 1m,n). These data indicate that IL‐6 can be an early inflammatory cytokine that may contribute to early hearing impairment in AHL.

### 3.2 Putative sources of IL‐6 and expression of IL‐6 receptor in the cochlea

Immunofluorescence was used to identify the putative source of IL‐6 in the cochlea to investigate the involvement of IL‐6 in AHL. It was observed that IL‐6 was highly enriched in SGNs and CD68‐positive macrophages in the lateral wall (Figure S1a,c) but not in hair cells (HCs) (Figure S1e). IL‐6 levels were also significantly elevated in SGNs and macrophages of 9‐month‐old WT mice (WT 9 M) compared with 2‐month‐old WT mice (WT 2 M) (Figure S1b,d). These results suggest that SGNs and macrophages contribute to IL‐6 secretion in the cochlea during AHL.

Next, we examined the expression of IL‐6 receptors in WT 2 M and WT 9 M mice. The IL‐6 receptor was abundantly expressed in hair cells, stria vascularis, and spiral ligament, but was absent in SGNs (Figure S1f–h).

### 3.3 IL‐6 deficiency protects against age‐related hearing loss in mice

**FIGURE 2:**
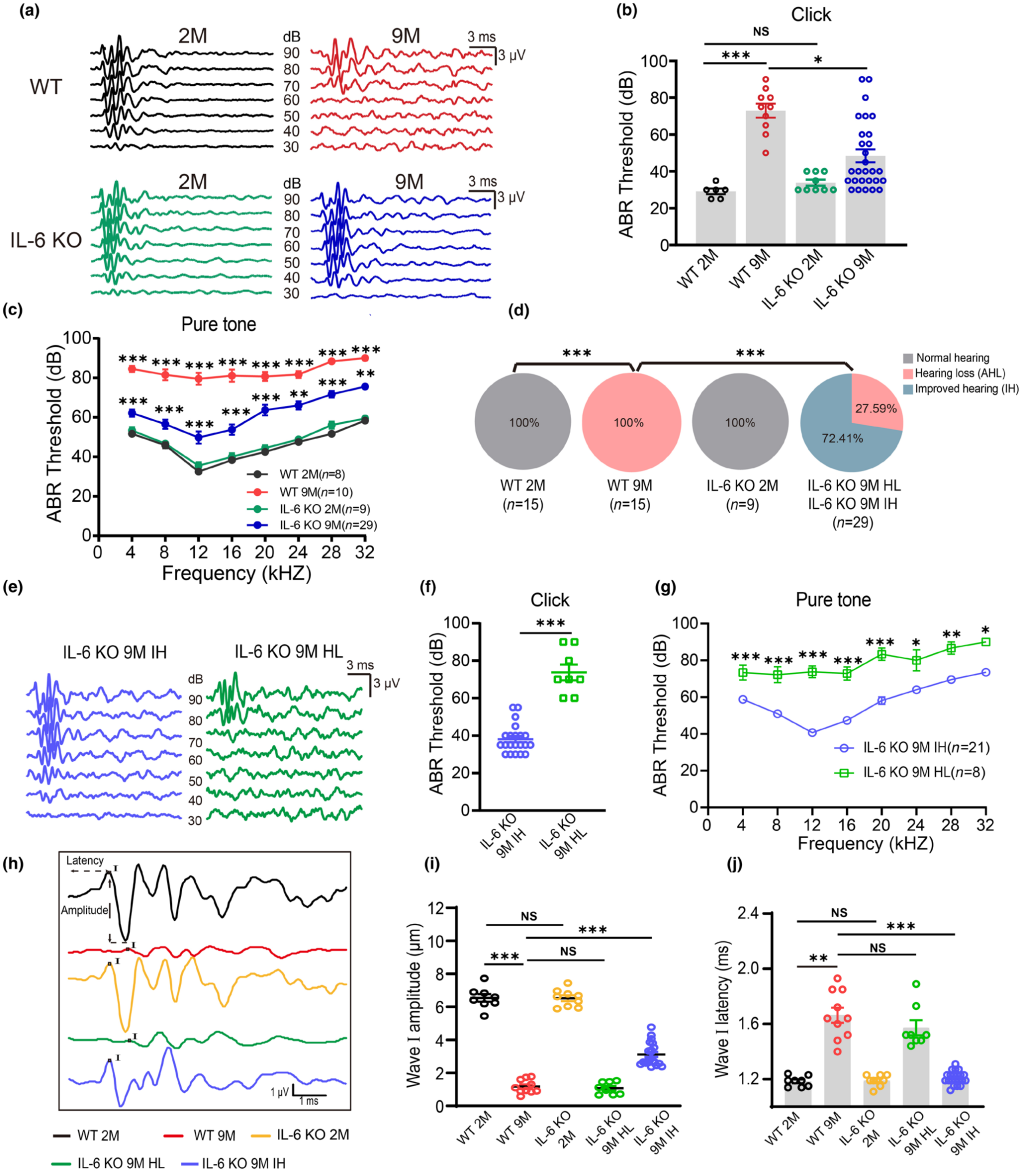
IL‐6 deficiency protects against age‐related hearing loss in mice. (a) Representative auditory brainstem response (ABR) waveforms in respond to clicking stimuli in WT 2 M, WT 9 M, IL‐6 KO 2 M, and IL‐6 KO 9 M mice. (b, c) Click values and ABR thresholds to pure‐tones were measured from WT 2 M, WT 9 M, IL‐6 KO 2 M, and IL‐6 KO 9 M mice. *versus WT 2 M, # versus WT 9 M in c. (d) Pie charts illustrating the percentage abundance of hearing function in WT and IL‐6 KO mice. (e) Representative ABR waveforms in respond to clicking 90 dB sound pressure level in IL‐6 KO 9 M mice with hearing loss (IL‐6 KO 9 M HL) and IL‐6 KO 9 M mice with hearing improved (IL‐6 KO 9 M IH). (f, g) Click values and ABR thresholds to pure‐tones in IL‐6 KO 9 M HL and IL‐6 KO 9 M IH mice. (h) Representative ABR waveform I in WT 2 M, WT 9 M, IL‐6 KO 2 M, IL‐6 KO 9 M HL, and IL‐6 KO 9 M IH mice. (i, j) Summary of amplitude (I) and latency (j) of ABR wave I in WT and IL‐6 KO mice. Data are means ± SEM, **p < 0.01, ***p < 0.001.

ABR thresholds were measured in WT and IL‐6 KO mice (Figure 2a–c) to evaluate the effect of IL‐6 on auditory function. The 9‐month‐old IL‐6 KO mice (IL‐6 KO 9 M) exhibited reduced hearing loss compared with age‐matched WT 9 M mice, indicating that IL‐6‐related inflammation may contribute to AHL (Figure 2b,c). Furthermore, 72.41% of IL‐6 KO 9 M mice showed improved hearing (IH), which formed the IL‐6 KO 9 M IH group (Figure 2d). In contrast, 27.59% of the IL‐6 KO 9 M mice still displayed hearing loss (HL) (the IL‐6 KO 9 M HL group) (Figure 2d). Significant differences in hearing thresholds between IL‐6 KO 9 M IH and IL‐6 KO 9 M HL mice were also observed (Figure 2e–g), guiding subsequent experiments to clarify whether IL‐6 plays a different role in AHL.

To further identify the potential impact of IL‐6 on hearing function, we analyzed the amplitude and latency of wave I (Figure 2h–j). The amplitude representing the sum of SGN activities innervating the IHC was significantly reduced in WT 9 M mice compared with WT 2 M mice, suggesting decreased auditory signaling. Conversely, IL‐6 KO 9 M IH mice exhibited higher wave I amplitude than WT 9 M mice (Figure 2i) indicating improved auditory signaling from IHCs to SGNs. Similarly, the latencies reflecting auditory nerve conduction velocity were prolonged in WT 9 M mice compared with WT 2 M mice (Figure 2j), suggesting that myelination of the auditory nerve may be altered in WT 9 M mice. Moreover, the latency of IL‐6 KO 9 M IH mice was shorter than that of WT 9 M mice (Figure 2j), suggesting that the absence of IL‐6 likely affects myelination.

Next, we investigated the potential involvement of IL‐6 in myelin damage of SGNs during AHL. We assessed changes in myelin basic protein (MBP), which is a major component of myelin, through immunostaining. Additionally, we analyzed myelin destruction by counting the number of SGNs with intact MBP^+^ myelin sheaths in both WT and IL‐6 KO mice. Our findings reveal that the proportion of intact MBP^+^ myelin sheaths was lower in the WT 9 M mice compared to WT 2 M mice (Figure S2a,b), suggesting the occurrence of myelin damage in AHL. In contrast, IL‐6 KO 9 M IH mice exhibited a higher proportion of intact MBP^+^ myelin sheaths than WT 9 M mice (Figure S2a,b), suggesting the reduction in myelin sheaths damage in IL‐6 KO 9 M IH mice. Taken together, these results indicate that AHL impairs auditory signal transmission from IHCs to SGNs, and that IL‐6 KO protection against hearing loss likely attenuating impairment of signaling.

### 3.4 IL‐6 deficiency decreased age‐related upregulation of Ca_v_1.3 channel in IHCs

**FIGURE 3:**
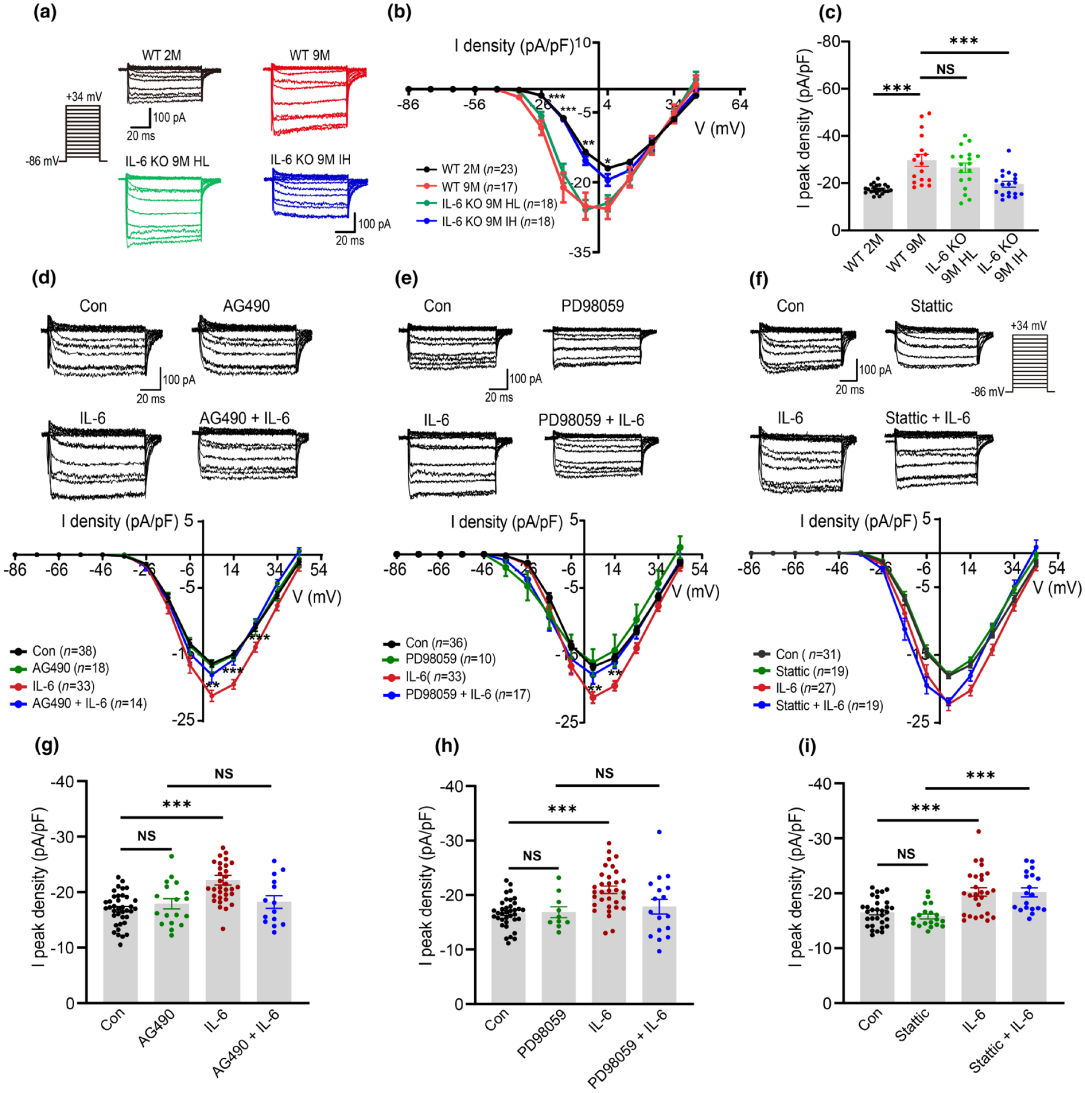
IL‐6 incudes upregulation of Ca_v_1.3 channel in IHCs via JAK‐MAPK pathway in vitro. (a) Representative traces of Ca^2+^ currents upon voltage step from −86 mV to +34 mV recorded from IHCs of WT 2 M, WT 9 M, IL‐6 KO 9 M HL, and IL‐6 KO 9 M IH mice, respectively. (b) Current density‐voltage curves obtained from IHCs of WT 2 M, WT 9 M, IL‐6 KO 9 M HL and IL‐6 KO 9 M IH mice. *IL‐6 KO 9 M IH versus WT 9 M. (c) Peak current density of IHCs in each group in panel b. (d–f) Effects of JAK inhibitor AG490 (d), MAPK inhibitor PD98059 (e) and STAT‐3 inhibitor Static (f) on Ca^2+^ currents after IL‐6 incubation. (g–i) Peak current density of IHCs in each group in panel d–f. Data are means ± SEM, *p < 0.05, **p < 0.01, ***p < 0.001 versus IL‐6.

To verify the role of IL‐6 in auditory signal transmission in AHL, we examined the effects of IL‐6 on ribbon synapses formed by IHCs and SGNs. Using the whole‐cell voltage‐clamp technique, we recorded Ca_v_1.3 channel currents at IHC ribbon synapses isolated from cochlear basilar membranes in WT and IL‐6 KO mice (Figure S3a). In WT 9 M mice, Ca_v_1.3 channel current density in IHCs exhibited a significant increase compared to WT 2 M mice (Figure 3a–c), suggesting an upregulation of Ca_v_1.3 in AHL. In contrast, IL‐6 KO 9 M IH mice displayed reduced Ca_v_1.3 channel current density in IHCs, while no significant difference was observed between IL‐6 KO 9 M HL mice and WT 9 M mice (Figure 3a–c).

We further analyzed the voltage dependence of the Ca_v_1.3 channel by fitting its activation curves to the Boltzmann equation. An increase in the half‐activation voltage (V_1/2_) and a decrease in the slope factor were observed in WT 9 M mice compared to WT 2 M mice (Figure S3b–d). Similarly, a significant difference was noted in the V_1/2_ and slope factors between IL‐6 KO 9 M IH mice and WT 9 M mice (Figure S3c,d). These results indicate that Ca_v_1.3 is normally upregulated in AHL and that KO of IL‐6 is associated with reduced age‐related upregulation of Ca_v_1.3.

### 3.5 IL‐6 upregulated the Ca_v_1.3 channel in IHCs via the Janus kinase‐mitogen activated kinase pathway in vitro

To verify the regulatory effect of IL‐6 on the Ca_v_1.3 channel, we recorded Ca_v_1.3 channel currents in WT IHCs incubated with IL‐6 (100 ng/mL). IL‐6 incubation for 30 and 60 min increased the Ca_v_1.3 channel current density in IHCs (Figure S4a,b). The V1/2 also increased significantly in IHCs treated with IL‐6 (Figure S4c,d), whereas the slope factor did not change (Figure S4e).

To identify which components of the signaling pathways were involved in IL‐6‐mediated upregulation of the Ca_v_1.3 channel in IHCs, we evaluated the effect of AG490 (10 μM, a Janus kinase (JAK) inhibitor), PD98059 (10 μM, a mitogen‐activated kinase (MAPK) inhibitor), and Stattic (20 μM, STAT3 inhibitor) on Ca_v_1.3 currents in IHCs induced by IL‐6, respectively. We found AG490 and PD98059 attenuated the IL‐6‐upregulated Ca_v_1.3 channel current density in IHCs (Figure 3d,e,g,h). Conversely, Stattic failed to inhibit the enhancement of Ca_v_1.3 channel currents in IL‐6‐treated cells (Figure 3f,i). These data indicate that IL‐6 upregulates the Ca_v_1.3 channel in IHCs via the JAK‐MAPK pathway.

In addition, our previous study showed that SGNs also express Ca_v_1.3 (Lv et al., 2012); therefore, we investigated whether IL‐6 has an effect on Ca^2+^ channels in SGNs by using the L‐type Ca^2+^ channel blocker nimodipine. No significant difference was observed in nimodipine‐sensitive currents between the control and the IL‐6 treatment group (Figure S5a–f), indicating the SGNs were not affected by IL‐6.

### 3.6 IL‐6 increased exocytosis in IHCs during AHL

**FIGURE 4:**
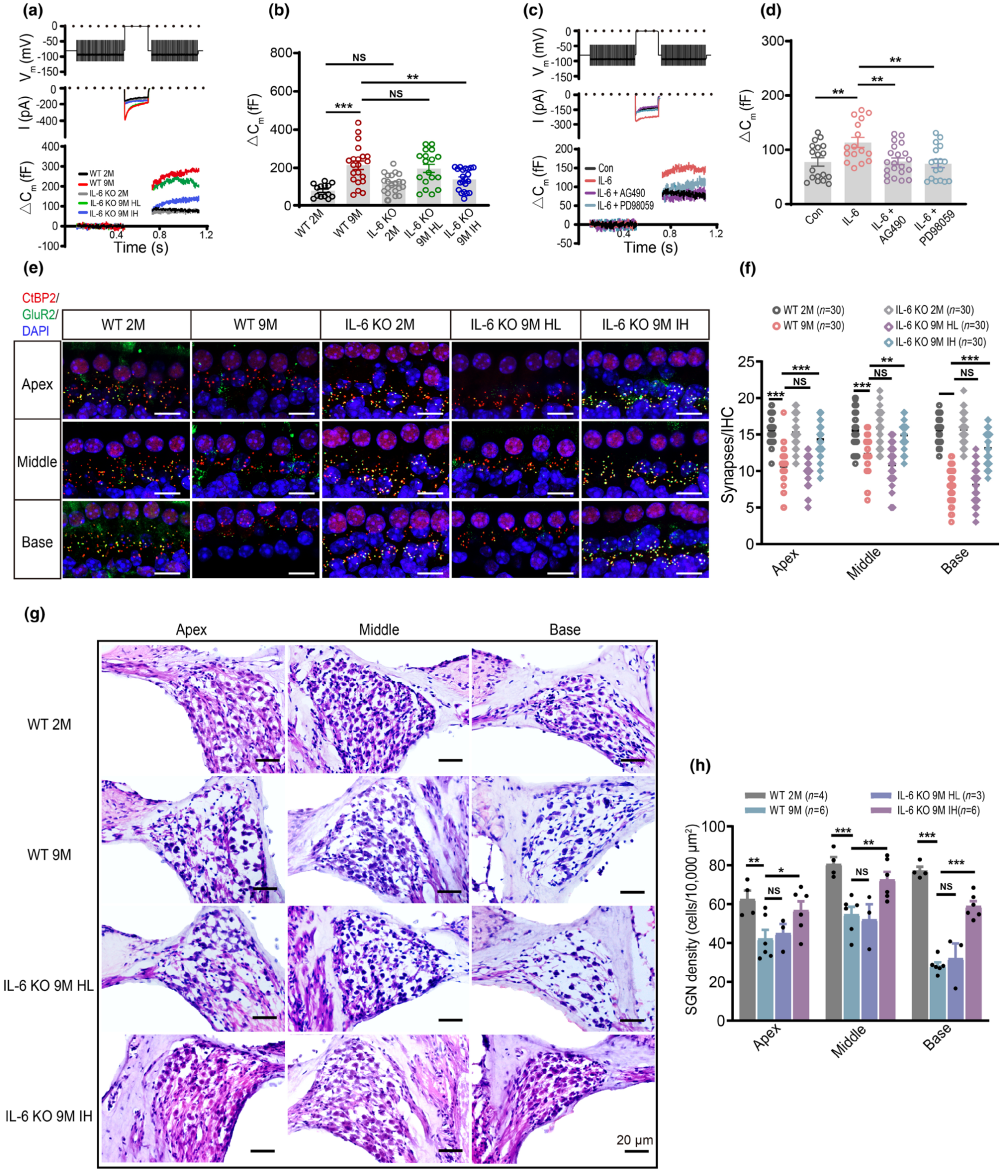
IL‐6 induces increase of exocytosis in IHCs and leads to damage of ribbon synapses and SGNs during AHL. (a) Representative traces of Ca^2+^ currents (middle row) and △Cm (bottom row) in IHCs from WT and IL‐6 KO mice. Recordings were obtained in a 200‐ms depolarization response from −80 mV to 0 mV (top row). (b) △Cm values recorded from IHCs of WT and IL‐6 KO mice. (c) Representative traces of Ca^2+^ currents and △Cm induced by IL‐6 in IHCs upon application of AG490 or PD98059 in vitro. (d) △Cm values recorded from IHCs in Con, IL‐6, IL‐6 + AG490, and IL‐6 + PD98059 groups. (e) Immunofluorescence of IHCs from apex, middle, and base of cochlea in WT and IL‐6 KO mice labeled with anti‐CtBP2 (red) and anti‐GluR2 (green). Scale bar: 10 μm (f) Quantification of ribbon synapses in IHCs from WT and IL‐6 KO mice. (g) Morphology of SGNs from apical, middle, and basal cochlea in WT and IL‐6 KO mice. Scale bar: 20 μm (h) Quantification of SGNs in apical, middle, and basal cochlea of WT and IL‐6 KO mice. Data are means ± SEM, *p < 0.05, **p < 0.01, ***p < 0.001.

To investigate whether IL‐6‐upregulated Ca_v_1.3 caused defects in vesicle exocytosis at IHC ribbon synapses, we measured ΔCm using whole‐cell patch‐clamp techniques in WT and IL‐6 KO mice. Figure 4a shows the Ca_v_1.3 channel currents (middle row) and the corresponding ΔCm (bottom row) recorded from IHCs of WT and IL‐6 KO mice using a 200‐ms depolarization response from −80 mV to 0 mV (top row). The ΔCm in IHC of WT AHL mice was higher than that of WT young mice, suggesting an enhancement in exocytosis at IHC ribbon synapses during AHL (Figure 4b). Consistent with the changes in Ca_v_1.3 channel currents (Figure 3b,c), the ΔCm was decreased in IHCs of IL‐6 KO IH mice compared to WT AHL mice (Figure 4b). These data suggest that upregulation of IL‐6 is associated with the increased release of neurotransmitters from IHCs during AHL.

We further verified whether IL‐6 enhanced exocytosis in IHCs via the JAK‐MAPK pathway. Incubation of IL‐6 (100 ng/mL) for 30 min significantly increased the ΔCm of IHCs (Figure 4c,d). AG490 (10 μM) or PD98059 (10 μM) attenuated the increase in ΔCm in IHCs, suggesting the JAK‐MAPK pathway may be involved in the IL‐6‐enhanced exocytosis.

Since enhanced exocytosis can potentially excessive glutamate release and subsequent excitotoxicity, we investigated whether it affects ribbon synapses, and SGNs during AHL. We first evaluated the number of ribbon synapses in the IHCs of WT and IL‐6 KO mice by labeling them with the presynaptic ribbon protein RIBEYE (CtBP2) and postsynaptic GluR2 (Figure 4e). The number of synapse puncta (CtBP2^+^, GluR2^+^) was significantly reduced in the IHCs of the cochlear apical, middle, and basal turns in WT 9 M mice (Figure 4f), suggesting ribbon synapse impairment during AHL. Furthermore, the loss of synapse puncta in IHCs was significantly reduced in IL‐6 KO 9 M IH mice but not in IL‐6 KO 9 M HL mice (Figure 4f). Moreover, no significant difference was observed between WT 2 M and IL‐6 KO 2 M mice (Figure 4f), indicating that IL‐6 deficiency did not affect the numbers of synapse in young mice. Subsequently, we examined SGN morphology in WT and IL‐6 KO mice (Figure 4g,h), noting that SGN loss was markedly reduced in IL‐6 KO 9 M IH mice (Figure 4h). Conversely, no difference in the number of SGNs was observed between IL‐6 KO 9 M HL and WT 9 M mice (Figure 4h).

### 3.7 L‐type Ca^2+^ channel blocker nimodipine rescued age‐related hearing loss

**FIGURE 5:**
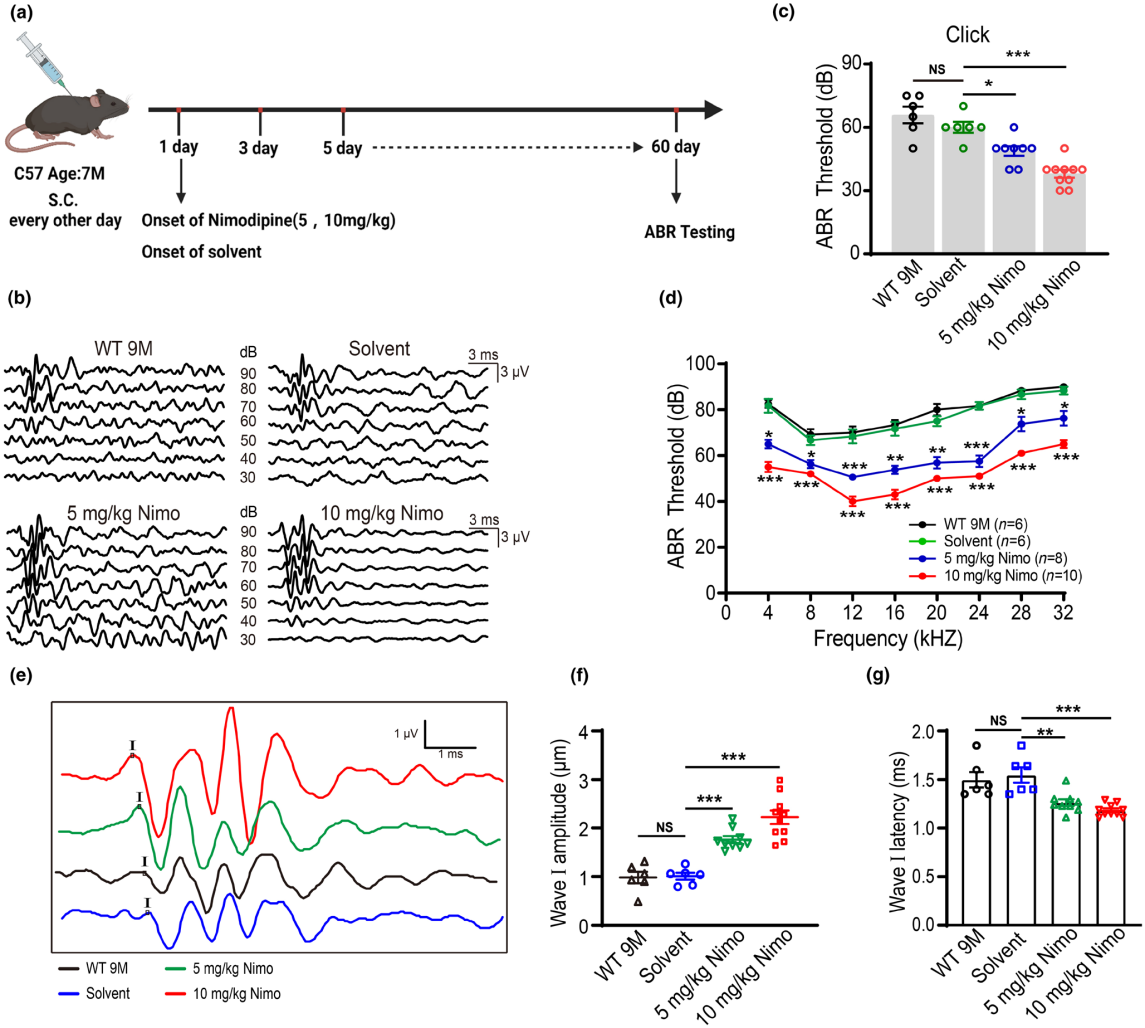
L‐type Ca^2+^ channel blocker nimodipine rescues age‐related hearing loss in mice. (a) Timeline for ABR testing and drug treatment. nimodipine was administered subcutaneously to 7‐month‐old WT mice at a dose of 5 mg/kg or 10 mg/kg every other day for 60 days. (b) Representative ABR waveforms in response to click stimuli (90–30 dB) in mice. (c, d) ABR threshold to click stimuli (c) and pure‐tone (d) in the AHL, solvent, and nimodipine (5, 10 mg/kg) treatment groups. * versus solvent. (e) Representative ABR waveforms in response to a click of 90 dB sound pressure level in mice. (f, g) Summary of amplitude (f) and latency (g) of ABR wave I in WT 9 M, solvent, and nimodipine treated mice. Data are means ± SEM, *p < 0.05, **p < 0.01, ***p < 0.001.

To confirm whether elevated Ca_v_1.3 channels contributes to AHL, we administered nimodipine (s.c., 5 and 10 mg/kg, every 2 days) to 7‐month‐old WT mice and evaluated their hearing function at 9 months of age (Figure 5a). The ABR thresholds for click (Figure 5b,c) and pure‐tone (Figure 5d) stimuli in WT 9 M mice were significantly reduced following the treatment with 5 and 10 mg/kg nimodipine. We further observed an increase in amplitude (Figure 5e,f) and a decrease in latency (Figure 5g) of ABR wave I in nimodipine‐treated mice, indicating protective effects on hearing during AHL.

**FIGURE 6:**
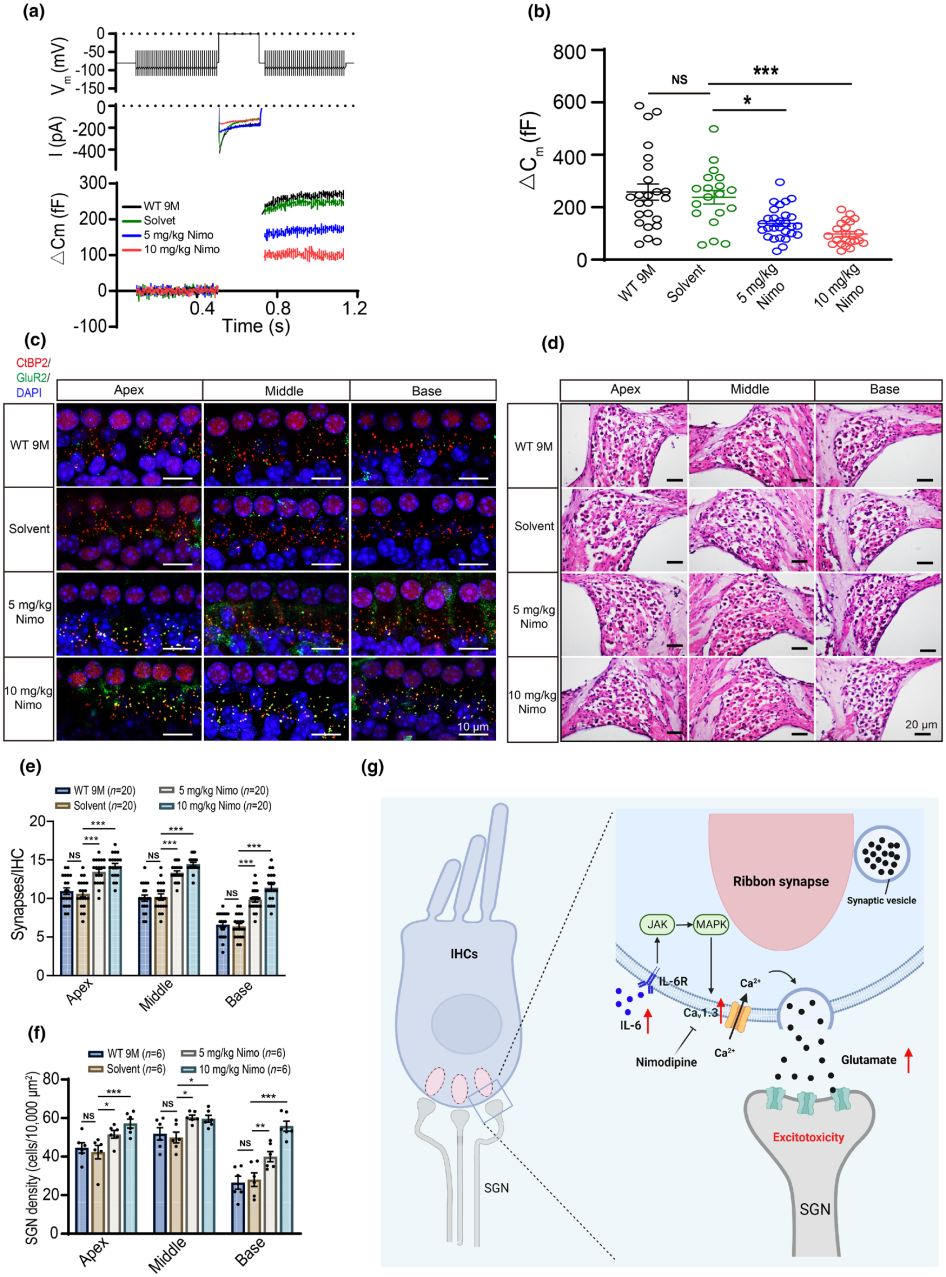
Nimodipine alleviates damage to ribbon synapses and SGNs. (a) Representative Ca^2+^ currents and △Cm of IHCs from WT 9 M mice, solvent‐treated, and nimodipine‐treated mice. (b) Summary of △Cm in IHCs of mice from WT 9 M, solvent, and nimodipine groups. (c) Immunofluorescence of IHCs and ribbon synapses in the apex, middle, and base of cochlea from WT 9 M mice, solvent‐treated, and nimodipine‐treated mice. Scale bar: 10 μm. (d) Morphological changes of SGNs in WT 9 M mice, solvent‐treated, and nimodipine‐treated mice. Scale bar: 20 μm. (e) Quantification of ribbon synapses in mice from different groups. (f) Quantification of SGNs in the cochlea's apical, middle, and basal regions of mice from different groups. Model for the mechanisms of AHL induced by upregulation of IL‐6. (g) The illustration demonstrates that increased expression of IL‐6 in the cochlea is associated with auditory dysfunction during AHL. IL‐6 upregulates Ca_v_1.3 channels via JAK‐MAPK pathway in the IHCs, which is involved in enhancing exocytosis and impairing ribbon synapses and the SGNs in AHL. Data are mean ± SEM, *p < 0.05, **p < 0.01, ***p < 0.001.

We subsequently determined whether nimodipine affects exocytosis by IHC and attenuates the impairment of ribbon synapses and SGNs during AHL. Nimodipine decreased the ΔCm in IHCs (Figure 6a,b) and increased the number of synapses in the apex, middle, and base of the cochlea (Figure 6c,e). Additionally, nimodipine inhibited the loss of SGNs in WT 9 M mice (Figure 6d,f). Thus, nimodipine is protective against age‐related cochlear damage.

Our results indicate that elevated IL‐6 expression in the cochlea is associated with auditory dysfunction during AHL. IL‐6 upregulates Ca_v_1.3 channels via JAK‐MAPK pathway in the IHCs and is involved in enhancing exocytosis and impairment of ribbon synapses and the SGNs in AHL (Figure 6g). IL‐6 deficiency or Ca_v_1.3 blockade were effective in rescuing AHL. Therefore, IL‐6‐dependent inflammaging, which upregulates Ca_v_1.3 channel in IHCs, play a significant role in contributing to AHL.

## 4 DISCUSSION

Identifying the molecular mechanisms underlying inflammaging in AHL is crucial for developing effective preventive strategies for hearing deficits. Our study revealed an unknown relationship between inflammaging and AHL, providing evidence that implicates IL‐6‐medicated inflammaging in early hearing dysfunction during AHL. IL‐6 upregulated Ca_v_1.3 calcium channels in IHCs, contributing to synapse impairment, SGNs degeneration, and hearing loss.

The body's immune function declines with age, leading to weakened immune surveillance and pathogen clearance (Yousefzadeh et al., 2021), which in turn causes a chronic inflammatory response known as inflammaging. The cochlea is thought to be an immune‐privileged organ because of the presence of a blood‐labyrinth barrier in the lateral wall (McCabe, 1989). However, several lines of evidence have revealed that the cochlea has resident macrophages and is vulnerable to systemic inflammation (Kampfe Nordstrom et al., 2018; Kishimoto et al., 2019; Miwa et al., 2024; Miwa & Okano, 2022). Inflammaging can induce bone marrow monocyte‐driven macrophages to infiltrate the cochlea and differentiate into senescent macrophages (Miwa & Okano, 2022). Aging cochlear macrophages were activated to produce pro‐inflammatory cytokines contributing to AHL (Frye et al., 2017; Noble et al., 2019). Several studies have explored how inflammation and immune cells in the inner ear contribute to AHL using C57BL/6 (C57) mice (Frye et al., 2017; Su et al., 2020). C57 mice display progressive loss of hair cells, degeneration of SGNs, the stria vascularis, and the spiral ligament, which aligns with the main characteristics of AHL (Schuknecht & Gacek, 1993). Therefore, it is widely used as an animal model for the AHL. However, unlike deafness caused by natural aging, C57 mice, resulting from mutations in the *Cdh23* gene, encode a component of the tip link in hair‐cell stereocilia, which has been identified as an important contributor to AHL (Johnson et al., 2000; Noben‐Trauth et al., 2003). To eliminate any interference caused by the mouse strain as a model of AHL, we also used IL‐6 KO mice on a C57 background, which exhibit normal development (Kopf et al., 1994), to investigate whether/how IL‐6‐related inflammaging contributes to hearing impairment in AHL.

Cochlear inflammation is a character of early‐onset aging‐related hearing loss in C57 mice (Frye et al., 2017). Similarly, we observed increased macrophage infiltration with high levels of IL‐6 in the cochlea of C57 mice with AHL. We found the macrophages were one of the major sources of IL‐6 in AHL, while IL‐6 immunoreactivity was also observed in SGNs, which could serve as an additional source of IL‐6. Furthermore, IL‐6 receptor was found in hair cells, stria vascularis, and spiral ligament but not in SGNs. These observations suggest that IL‐6 may act on various target cells in the cochlea. To further explore the role of IL‐6 in AHL, we utilized IL‐6 KO mice. We observed that 2‐month‐old IL‐6 KO mice exhibited normal hearing ability, whereas hearing variation was observed in 9‐month‐old IL‐6 KO mice. Cochlear inflammation associated with hearing loss was evident in 9‐month‐old C57 mice, while IL‐6 deficiency significantly attenuated this hearing loss. This suggests that IL‐6 may contribute to cochlear inflammation and play an important role in early hearing loss during AHL.

In rodents, ABR wave I represents the transduction of auditory signals from IHCs to SGNs. Reduced amplitudes and prolonged latencies of wave I were observed in mice with AHL, suggesting abnormalities in hearing sensitivity, and functional integrity encoded by ribbon synapses. Damage to synaptic connections affects the normal encoding of the temporal properties of sound, especially in noisy environments (Costalupes et al., 1984). During aging, ribbon synapse loss is accompanied by degeneration of the corresponding central auditory nerve synapses, which affects the signal processing of the entire central auditory system, leading to hearing deficit (Wang et al., 2021). In this study, we demonstrated that the synaptic machinery of IHCs in WT 9 M mice underwent several changes, including upregulated Ca_v_1.3 channels, enhanced exocytosis, and a reduction in ribbons. Moreover, recent research has noted that remaining ribbons in aged IHCs increase in volume (Jeng et al., 2020b), suggesting that the larger synaptic ribbons may accommodate more synaptic vesicles (Stamataki et al., 2006); containing Ca^2+^‐dependent glutamate, despite their reduced number in aged IHCs.

Glutamate release and reuptake are tightly regulated to optimize rapid synaptic transmission and maintain homeostasis. Glutamate over‐release leads to excitotoxicity and eventually SGN degeneration (Ding et al., 2021); a phenomenon supported by our findings of enhanced synaptic exocytosis and reduced SGN numbers in WT 9 M mice. These observations highlight the critical role of Ca_v_1.3 channels in neurotransmission within IHCs, where changes in cell membrane capacitance (ΔCm) serves as a pivotal marker of neurotransmitter release from presynaptic cells (Johnson et al., 2005; Moser & Beutner, 2000). To investigate whether IL‐6‐upregulated Ca_v_1.3 caused defects in vesicle exocytosis at IHC ribbon synapses, we measured the level of ΔCm in vivo and in vitro. The ΔCm in IHC of WT 9 M mice was enhanced, which is attenuated by IL‐6 deficiency. IL‐6 enhanced ΔCm in IHCs via the JAK‐MAPK pathway in vitro. These data suggest that upregulation of IL‐6 is associated with the increased release of neurotransmitters from IHCs during AHL.

In this study, it was observed that blockade of Ca_v_1.3 channels with nimodipine mitigated over‐exocytosis and preserved ribbon synapses and SGN in AHL. These results support that upregulation of Ca_v_1.3 channel by IL‐6 in IHCs may be an important contributing factor for AHL. However, hearing loss was not alleviated in 27.59% of IL‐6 KO 9 M mice (IL‐6 KO 9 M HL) in the present study. These mice exhibited increased Ca_v_1.3 channel currents, enhanced exocytosis, and reduced synapses numbers in IHCs compared to the “improved hearing” IL‐6 KO 9 M IH mice, suggesting that Ca_v_1.3 upregulation may play an important role in deafness. KO of IL‐6 in some mice model (IL‐6 KO 9 M HL) did not inhibit Ca_v_1.3 upregulation, indicating that other mechanisms, apart from IL‐6‐dependent pathways may also be involved in the regulation of Ca_v_1.3 in AHL, warranting further investigation.

In summary, our data revealed that IL‐6‐dependent inflammaging plays a crucial role in the onset of AHL. For the first time, we demonstrated that IL‐6 up‐regulates Ca_v_1.3 channels through the JAK‐MAPK pathway, contributing to neurotransmitter excitotoxicity and synaptic impairment in the cochlea of mice with AHL. IL‐6 deficiency or a Ca_v_1.3 channel blocker attenuated this damage, rescuing hearing loss. This finding uncovers a mechanism linking inflammation to synaptic damage, offering a new intervention strategy to mitigate the progression of AHL.

## AUTHOR CONTRIBUTIONS

PL and SH designed the research, wrote, reviewed, and edited the manuscript; ML, FX, JX, GH, SW, XL, HY, HL, HZ, YY, and XJ performed the research and analyzed the data. XF, HD and YL reviewed and edited the manuscript.

## CONFLICT OF INTEREST STATEMENT

The authors declare no conflict of interest.

## Supporting information


**Figure S1.** Expression of IL‐6 and IL‐6R in the cochlea of WT 2 M and WT 9 M mice. (a) Expression of IL‐6 in SGNs from WT 2 M and WT 9 M mice. Spiral ganglion neurons (SGNs) are labeled with anti‐TUJ1, a neuron marker (green), and anti‐IL‐6 (red). (b) Quantification of IL‐6 in SGNs of WT 2 M and WT 9 M mice. (c) Expression of IL‐6 (red) and CD68 (green) in lateral wall of cochlea from WT 2 M and WT 9 M mice. (d) Quantification of IL‐6 in macrophages of WT 2 M and WT 9 M mice. (e) Expression of IL‐6 in hair cells of WT 2 M and WT 9 M mice. Hair cells are labeled with anti‐Myo7a (green) and anti‐IL‐6 (red). (f–h) Expression of IL‐6R in SGNs (f), stria vascularis and spiral ligament (g) and hair cells (h) in WT 2 M and WT 9 M mice. Scale bar: 25 μm in a, c and e–h. Data are means ± SEM, ***p < 0.001.

**Figure S2.** Changes in MBP expression in SGNs of WT and IL‐6 KO mice. **(**a) Expression of MBP in SGNs from WT 2 M, WT 9 M, IL‐6 KO 2 M, IL‐6 KO 9 M HL, and IL‐6 KO 9 M IH mice. SGNs are labeled with anti‐MBP (red) and anti‐TUJ1, a neuron marker (green). Scale bar: 20 μm. (b) Quantitative analysis of SGNs with intact MBP^+^ myelin sheaths in WT and IL‐6 KO mice. Data are means ± SEM, ***p < 0.001.

**Figure S3.** IL‐6 induces upregulation of Ca_v_1.3 channel of IHCs in AHL. (a) Photomicrograph showing a row of IHCs and a patch‐clamp recording electrode allowing measurements of ionic currents. (b) Activation curves of Ca^2+^ channels recorded in IHCs from WT and IL‐6 KO mice. (c, d) Summaries of V_1/2_ and slope factor of activation curve in IHCs from WT and IL‐6 KO mice. Data are means ± SEM, *p < 0.05, ***p < 0.001.

**Figure S4.** IL‐6 incubation induces upregulation of Ca_v_1.3 channel in IHCs in vitro. (a) Representative traces of Ca^2+^ currents in IHCs after incubation of IL‐6 (100 ng/mL) for 30 min, 60 min, and 90 min. (b) Current density‐voltage curves obtained from IHCs in the control and IL‐6 incubated groups. *30 min, 60 min versus Control. (c) Activation curves of Ca^2+^ channels fitted with the Boltzmann equation in IHCs. (d, e) V_1/2_ and slop factor of activation curve in IHCs of control and IL‐6 30 min groups.

**Figure S5.** IL‐6 incubation does not upregulate of nimodipine‐sensitive Ca^2+^ currents in SGNs in vitro. (a, b) Current density–voltage curves obtained from apical SGNs before and after application of nimodipine in control and IL‐6 treatment (incubated with IL‐6 at 20 ng/mL for 12 h). (c) The nimodipine‐sensitive Ca^2+^ currents in apical SGNs in control and IL‐6 treated groups. (d, e) Current density–voltage curves obtained from basal SGNs before and after application of nimodipine in control and IL‐6 treatment. (f) The nimodipine‐sensitive Ca^2+^ currents in basal SGNs in control and IL‐6 treated groups.

Table S1.

Table S2.

## Data Availability

All data generated or analyzed during this study were included in the manuscript.

## References

[acel14305-bib-0001] Baylis, D. , Bartlett, D. B. , Syddall, H. E. , Ntani, G. , Gale, C. R. , Cooper, C. , Lord, J. M. , & Sayer, A. A. (2013). Immune‐endocrine biomarkers as predictors of frailty and mortality: A 10‐year longitudinal study in community‐dwelling older people. Age (Dordrecht, Netherlands), 35, 963–971. 22388931 10.1007/s11357-012-9396-8 PMC3636387

[acel14305-bib-0002] Blasko, I. , Stampfer‐Kountchev, M. , Robatscher, P. , Veerhuis, R. , Eikelenboom, P. , & Grubeck‐Loebenstein, B. (2004). How chronic inflammation can affect the brain and support the development of Alzheimer's disease in old age: The role of microglia and astrocytes. Aging Cell, 3, 169–176. 15268750 10.1111/j.1474-9728.2004.00101.x

[acel14305-bib-0003] Costalupes, J. A. , Young, E. D. , & Gibson, D. J. (1984). Effects of continuous noise backgrounds on rate response of auditory nerve fibers in cat. Journal of Neurophysiology, 51, 1326–1344. 6737033 10.1152/jn.1984.51.6.1326

[acel14305-bib-0004] Ding, D. , Qi, W. , Jiang, H. , & Salvi, R. (2021). Excitotoxic damage to auditory nerve afferents and spiral ganglion neurons is correlated with developmental upregulation of AMPA and KA receptors. Hearing Research, 411, 108358. 34607211 10.1016/j.heares.2021.108358

[acel14305-bib-0005] Franceschi, C. , Garagnani, P. , Parini, P. , Giuliani, C. , & Santoro, A. (2018). Inflammaging: A new immune‐metabolic viewpoint for age‐related diseases. Nature Reviews. Endocrinology, 14, 576–590. 10.1038/s41574-018-0059-4 30046148

[acel14305-bib-0006] Frye, M. D. , Yang, W. , Zhang, C. , Xiong, B. , & Hu, B. H. (2017). Dynamic activation of basilar membrane macrophages in response to chronic sensory cell degeneration in aging mouse cochleae. Hearing Research, 344, 125–134. 27837652 10.1016/j.heares.2016.11.003 PMC5239751

[acel14305-bib-0007] GBD 2019 Hearing Loss Collaborators (2021) . (2021). Hearing loss prevalence and years lived with disability, 1990‐2019: Findings from the global burden of disease study 2019. Lancet, 397, 996–1009. 33714390 10.1016/S0140-6736(21)00516-X PMC7960691

[acel14305-bib-0008] Grant, R. W. , & Dixit, V. D. (2013). Mechanisms of disease: Inflammasome activation and the development of type 2 diabetes. Frontiers in Immunology, 4, 50. 23483669 10.3389/fimmu.2013.00050 PMC3592198

[acel14305-bib-0009] Jeng, J. Y. , Ceriani, F. , Hendry, A. , Johnson, S. L. , Yen, P. , Simmons, D. D. , Kros, C. J. , & Marcotti, W. (2020). Hair cell maturation is differentially regulated along the tonotopic axis of the mammalian cochlea. The Journal of Physiology, 598, 151–170. 31661723 10.1113/JP279012 PMC6972525

[acel14305-bib-0010] Jeng, J. Y. , Ceriani, F. , Olt, J. , Brown, S. D. M. , Holley, M. C. , Bowl, M. R. , Johnson, S. L. , & Marcotti, W. (2020). Pathophysiological changes in inner hair cell ribbon synapses in the ageing mammalian cochlea. The Journal of Physiology, 598, 4339–4355. 32710572 10.1113/JP280018 PMC7612121

[acel14305-bib-0011] Johnson, K. R. , Zheng, Q. Y. , & Erway, L. C. (2000). A major gene affecting age‐related hearing loss is common to at least ten inbred strains of mice. Genomics, 70, 171–180. 11112345 10.1006/geno.2000.6377

[acel14305-bib-0012] Johnson, S. L. , Marcotti, W. , & Kros, C. J. (2005). Increase in efficiency and reduction in Ca2+ dependence of exocytosis during development of mouse inner hair cells. The Journal of Physiology, 563, 177–191. 15613377 10.1113/jphysiol.2004.074740 PMC1665557

[acel14305-bib-0013] Kampfe Nordstrom, C. , Danckwardt‐Lilliestrom, N. , Laurell, G. , Liu, W. , & Rask‐Andersen, H. (2018). The human endolymphatic sac and inner ear immunity: Macrophage interaction and molecular expression. Frontiers in Immunology, 9, 3181. 30774637 10.3389/fimmu.2018.03181 PMC6367985

[acel14305-bib-0014] Kishimoto, I. , Okano, T. , Nishimura, K. , Motohashi, T. , & Omori, K. (2019). Early development of resident macrophages in the mouse cochlea depends on yolk sac hematopoiesis. Frontiers in Neurology, 10, 1115. 31695671 10.3389/fneur.2019.01115 PMC6817595

[acel14305-bib-0015] Kopf, M. , Baumann, H. , Freer, G. , Freudenberg, M. , Lamers, M. , Kishimoto, T. , Zinkernagel, R. , Bluethmann, H. , & Kohler, G. (1994). Impaired immune and acute‐phase responses in interleukin‐6‐deficient mice. Nature, 368, 339–342. 8127368 10.1038/368339a0

[acel14305-bib-0016] Lindau, M. , & Neher, E. (1988). Patch‐clamp techniques for time‐resolved capacitance measurements in single cells. Pflügers Archiv, 411, 137–146. 3357753 10.1007/BF00582306

[acel14305-bib-0017] Livingston, G. , Huntley, J. , Sommerlad, A. , Ames, D. , Ballard, C. , Banerjee, S. , Brayne, C. , Burns, A. , Cohen‐Mansfield, J. , Cooper, C. , Costafreda, S. G. , Dias, A. , Fox, N. , Gitlin, L. N. , Howard, R. , Kales, H. C. , Kivimaki, M. , Larson, E. B. , Ogunniyi, A. , … Mukadam, N. (2020). Dementia prevention, intervention, and care: 2020 report of the lancet commission. Lancet, 396, 413–446. 32738937 10.1016/S0140-6736(20)30367-6 PMC7392084

[acel14305-bib-0018] Lv, P. , Sihn, C. R. , Wang, W. , Shen, H. , Kim, H. J. , Rocha‐Sanchez, S. M. , & Yamoah, E. N. (2012). Posthearing Ca(2+) currents and their roles in shaping the different modes of firing of spiral ganglion neurons. The Journal of Neuroscience, 32, 16314–16330. 23152615 10.1523/JNEUROSCI.2097-12.2012 PMC3535314

[acel14305-bib-0019] Lv, P. , Wei, D. , & Yamoah, E. N. (2010). Kv7‐type channel currents in spiral ganglion neurons: Involvement in sensorineural hearing loss. The Journal of Biological Chemistry, 285, 34699–34707. 20739290 10.1074/jbc.M110.136192 PMC2966085

[acel14305-bib-0020] Lyu, A. R. , Kim, T. H. , Park, S. J. , Shin, S. A. , Jeong, S. H. , Yu, Y. , Huh, Y. H. , Je, A. R. , Park, M. J. , & Park, Y. H. (2020). Mitochondrial damage and necroptosis in aging cochlea. International Journal of Molecular Sciences, 21, 2505. 32260310 10.3390/ijms21072505 PMC7177801

[acel14305-bib-0021] McCabe, B. F. (1989). Autoimmune inner ear disease: Therapy. The American Journal of Otology, 10, 196–197. 2750868

[acel14305-bib-0022] Miwa, T. , & Okano, T. (2022). Role of inner ear macrophages and autoimmune/autoinflammatory mechanisms in the pathophysiology of inner ear disease. Frontiers in Neurology, 13, 861992. 35463143 10.3389/fneur.2022.861992 PMC9019483

[acel14305-bib-0023] Miwa, T. , Rengasamy, G. , Liu, Z. , Ginhoux, F. , & Okano, T. (2024). Contribution of circulating monocytes in maintaining homeostasis of resident macrophages in postnatal and young adult mouse cochlea. Scientific Reports, 14, 62. 38167979 10.1038/s41598-023-50634-y PMC10762055

[acel14305-bib-0024] Moser, T. , & Beutner, D. (2000). Kinetics of exocytosis and endocytosis at the cochlear inner hair cell afferent synapse of the mouse. Proceedings of the National Academy of Sciences of the United States of America, 97, 883–888. 10639174 10.1073/pnas.97.2.883 PMC15425

[acel14305-bib-0025] Noben‐Trauth, K. , Zheng, Q. Y. , & Johnson, K. R. (2003). Association of cadherin 23 with polygenic inheritance and genetic modification of sensorineural hearing loss. Nature Genetics, 35, 21–23. 10.1038/ng1226 PMC2864026 12910270

[acel14305-bib-0026] Noble, K. V. , Liu, T. , Matthews, L. J. , Schulte, B. A. , & Lang, H. (2019). Age‐related changes in immune cells of the human cochlea. Frontiers in Neurology, 10, 895. 31474935 10.3389/fneur.2019.00895 PMC6707808

[acel14305-bib-0027] Osiecki, H. (2004). The role of chronic inflammation in cardiovascular disease and its regulation by nutrients. Alternative Medicine Review, 9, 32–53. 15005643

[acel14305-bib-0028] Platzer, J. , Engel, J. , Schrott‐Fischer, A. , Stephan, K. , Bova, S. , Chen, H. , Zheng, H. , & Striessnig, J. (2000). Congenital deafness and sinoatrial node dysfunction in mice lacking class D L‐type Ca2+ channels. Cell, 102, 89–97. 10929716 10.1016/s0092-8674(00)00013-1

[acel14305-bib-0029] Rutherford, B. R. , Brewster, K. , Golub, J. S. , Kim, A. H. , & Roose, S. P. (2018). Sensation and psychiatry: Linking age‐related hearing loss to late‐life depression and cognitive decline. The American Journal of Psychiatry, 175, 215–224. 29202654 10.1176/appi.ajp.2017.17040423 PMC5849471

[acel14305-bib-0030] Schuknecht, H. F. , & Gacek, M. R. (1993). Cochlear pathology in presbycusis. The Annals of Otology, Rhinology, and Laryngology, 102, 1–16. 10.1177/00034894931020S101 8420477

[acel14305-bib-0031] Seicol, B. J. , Lin, S. , & Xie, R. (2022). Age‐related hearing loss is accompanied by chronic inflammation in the cochlea and the Cochlear nucleus. Frontiers in Aging Neuroscience, 14, 846804. 35418849 10.3389/fnagi.2022.846804 PMC8995794

[acel14305-bib-0032] Sergeyenko, Y. , Lall, K. , Liberman, M. C. , & Kujawa, S. G. (2013). Age‐related cochlear synaptopathy: An early‐onset contributor to auditory functional decline. The Journal of Neuroscience, 33, 13686–13694. 23966690 10.1523/JNEUROSCI.1783-13.2013 PMC3755715

[acel14305-bib-0033] Shen, H. , Liu, W. , Geng, Q. , Li, H. , Lu, M. , Liang, P. , Zhang, B. , Yamoah, E. N. , & Lv, P. (2018). Age‐dependent up‐regulation of HCN channels in spiral ganglion neurons coincide with hearing loss in mice. Frontiers in Aging Neuroscience, 10, 353. 30459593 10.3389/fnagi.2018.00353 PMC6232381

[acel14305-bib-0034] Stamataki, S. , Francis, H. W. , Lehar, M. , May, B. J. , & Ryugo, D. K. (2006). Synaptic alterations at inner hair cells precede spiral ganglion cell loss in aging C57BL/6J mice. Hearing Research, 221, 104–118. 17005343 10.1016/j.heares.2006.07.014

[acel14305-bib-0035] Su, Z. , Xiong, H. , Liu, Y. , Pang, J. , Lin, H. , Zhang, W. , & Zheng, Y. (2020). Transcriptomic analysis highlights cochlear inflammation associated with age‐related hearing loss in C57BL/6 mice using next generation sequencing. PeerJ, 8, e9737. 32879802 10.7717/peerj.9737 PMC7443093

[acel14305-bib-0036] Verschuur, C. A. , Dowell, A. , Syddall, H. E. , Ntani, G. , Simmonds, S. J. , Baylis, D. , Gale, C. R. , Walsh, B. , Cooper, C. , Lord, J. M. , & Sayer, A. A. (2012). Markers of inflammatory status are associated with hearing threshold in older people: Findings from the Hertfordshire ageing study. Age and Ageing, 41, 92–97. 22086966 10.1093/ageing/afr140

[acel14305-bib-0037] Wang, M. , Zhang, C. , Lin, S. , Wang, Y. , Seicol, B. J. , Ariss, R. W. , & Xie, R. (2021). Biased auditory nerve central synaptopathy is associated with age‐related hearing loss. The Journal of Physiology, 599, 1833–1854. 33450070 10.1113/JP281014 PMC8197675

[acel14305-bib-0038] Wu, T. , Zhou, J. , Qiu, J. , Song, Y. , Guo, W. , Cui, L. , Song, X. , & Sun, Y. (2022). Tumor necrosis factor‐alpha mediated inflammation versus apoptosis in age‐related hearing loss. Frontiers in Aging Neuroscience, 14, 956503. 36158549 10.3389/fnagi.2022.956503 PMC9491822

[acel14305-bib-0039] Xiong, W. , Yu, S. , Liu, K. , & Gong, S. (2020). Loss of cochlear ribbon synapses in the early stage of aging causes initial hearing impairment. American Journal of Translational Research, 12, 7354–7366. 33312372 PMC7724364

[acel14305-bib-0040] Yousefzadeh, M. J. , Flores, R. R. , Zhu, Y. , Schmiechen, Z. C. , Brooks, R. W. , Trussoni, C. E. , Cui, Y. , Angelini, L. , Lee, K. A. , McGowan, S. J. , Burrack, A. L. , Wang, D. , Dong, Q. , Lu, A. , Sano, T. , O'Kelly, R. D. , McGuckian, C. A. , Kato, J. I. , M.P. Bank , … Niedernhofer, L. J. (2021). An aged immune system drives senescence and ageing of solid organs. Nature, 594, 100–105. 33981041 10.1038/s41586-021-03547-7 PMC8684299

