# Supplementary figures and images for: Upregulation of the Cav1.3 channel in inner hair cells by interleukin 6‐dependent inflammaging contributes to age‐related hearing loss
Source: Aging Cell. 2024 Aug 15;23(12):e14305. doi: 10.1111/acel.14305 (PMC11634703; doi:10.1111/acel.14305)

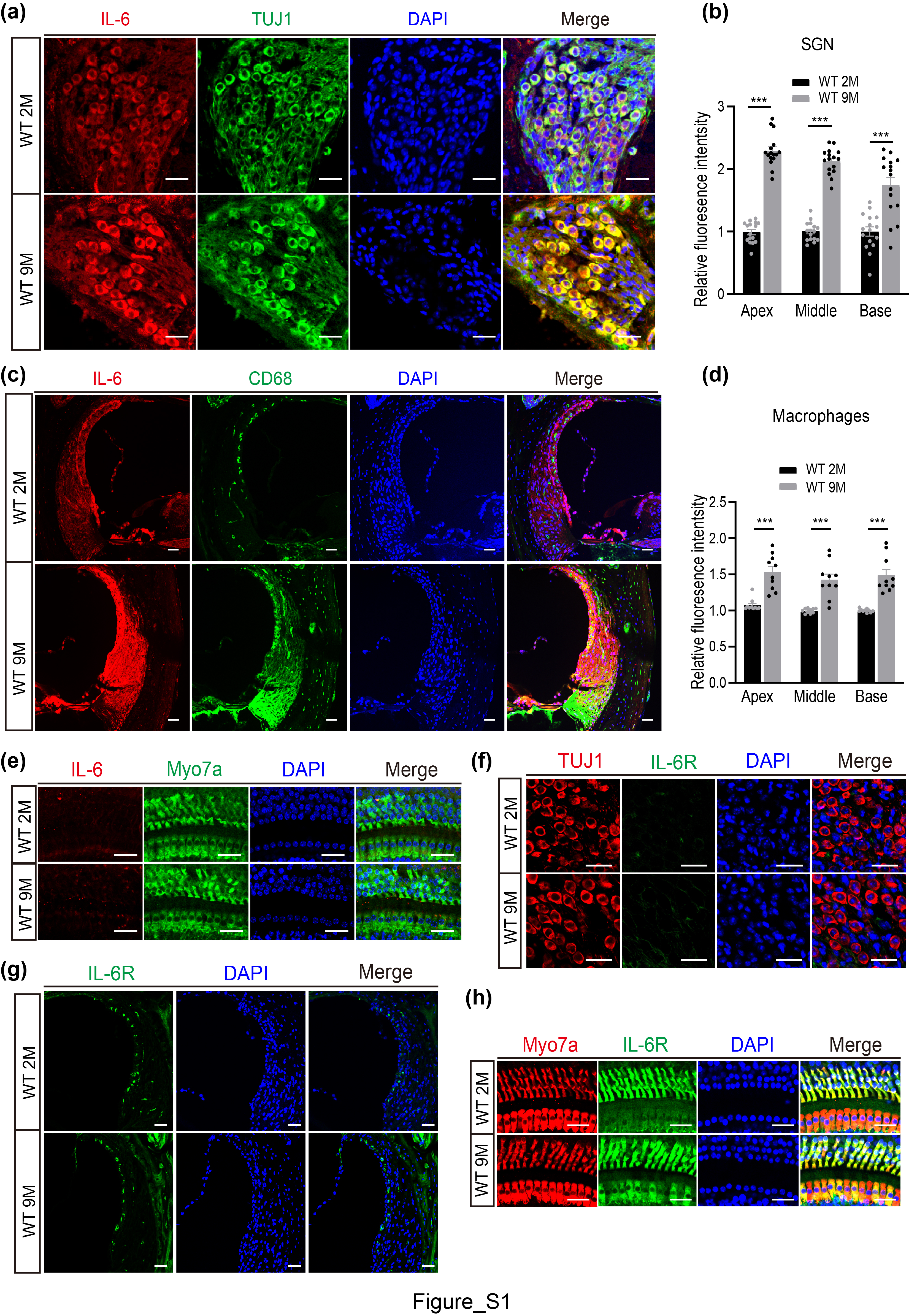

Supplement: Supplementary file 1 — Figure S1. Expression of IL‐6 and IL‐6R in the cochlea of WT 2 M and WT 9 M mice. (a) Expression of IL‐6 in SGNs from WT 2 M and WT 9 M mice. Spiral ganglion neurons (SGNs) are labeled with anti‐TUJ1, a neuron marker (green), and anti‐IL‐6 (red). (b) Quantification of IL‐6 in SGNs of WT 2 M and WT 9 M mice. (c) Expression of IL‐6 (red) and CD68 (green) in lateral wall of cochlea from WT 2 M and WT 9 M mice. (d) Quantification of IL‐6 in macrophages of WT 2 M and WT 9 M mice. (e) Expression of IL‐6 in hair cells of WT 2 M and WT 9 M mice. Hair cells are labeled with anti‐Myo7a (green) and anti‐IL‐6 (red). (f–h) Expression of IL‐6R in SGNs (f), stria vascularis and spiral ligament (g) and hair cells (h) in WT 2 M and WT 9 M mice. Scale bar: 25 μm in a, c and e–h. Data are means ± SEM, ***p < 0.001. [file ACEL-23-e14305-s004.jpg]

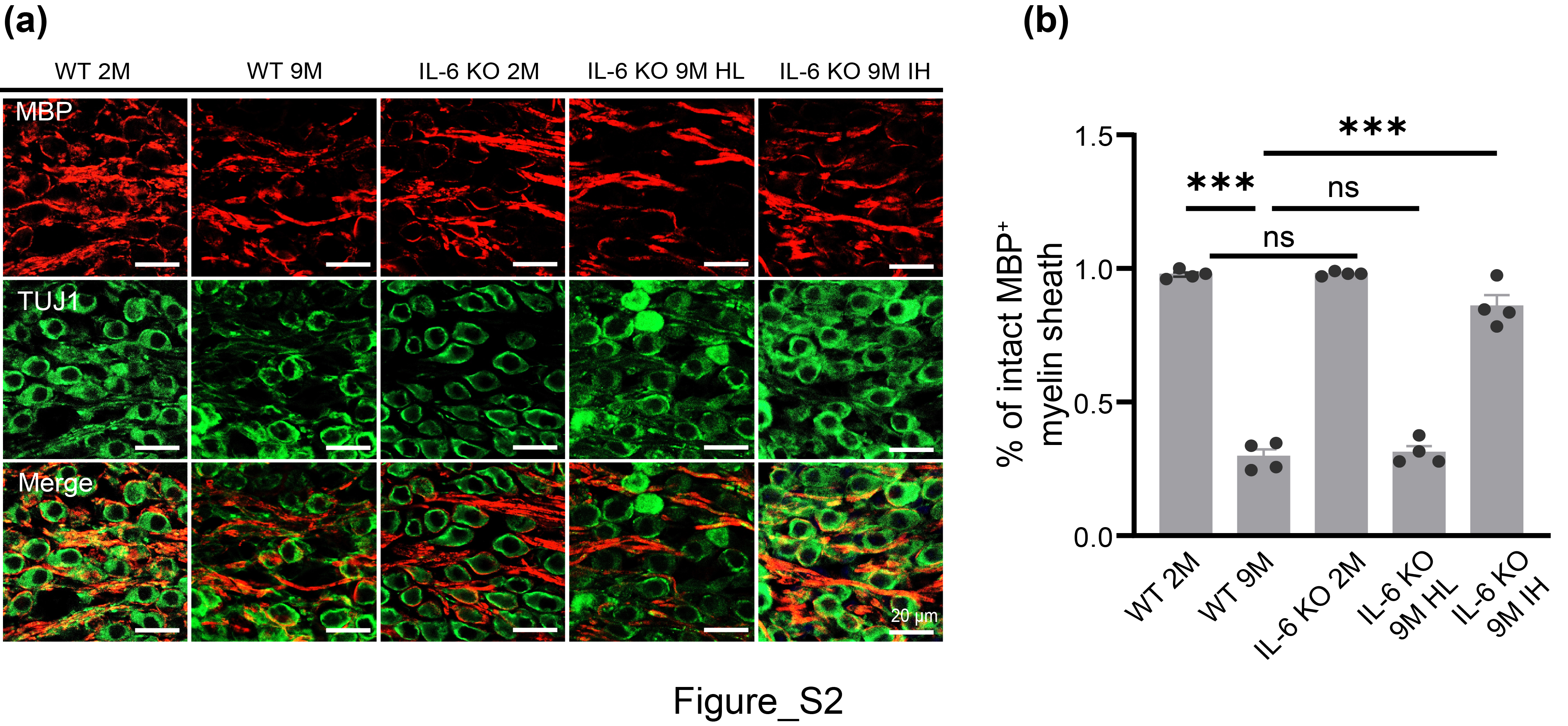

Supplement: Supplementary file 2 — Figure S2. Changes in MBP expression in SGNs of WT and IL‐6 KO mice. (a) Expression of MBP in SGNs from WT 2 M, WT 9 M, IL‐6 KO 2 M, IL‐6 KO 9 M HL, and IL‐6 KO 9 M IH mice. SGNs are labeled with anti‐MBP (red) and anti‐TUJ1, a neuron marker (green). Scale bar: 20 μm. (b) Quantitative analysis of SGNs with intact MBP+ myelin sheaths in WT and IL‐6 KO mice. Data are means ± SEM, ***p < 0.001. [file ACEL-23-e14305-s003.jpg]

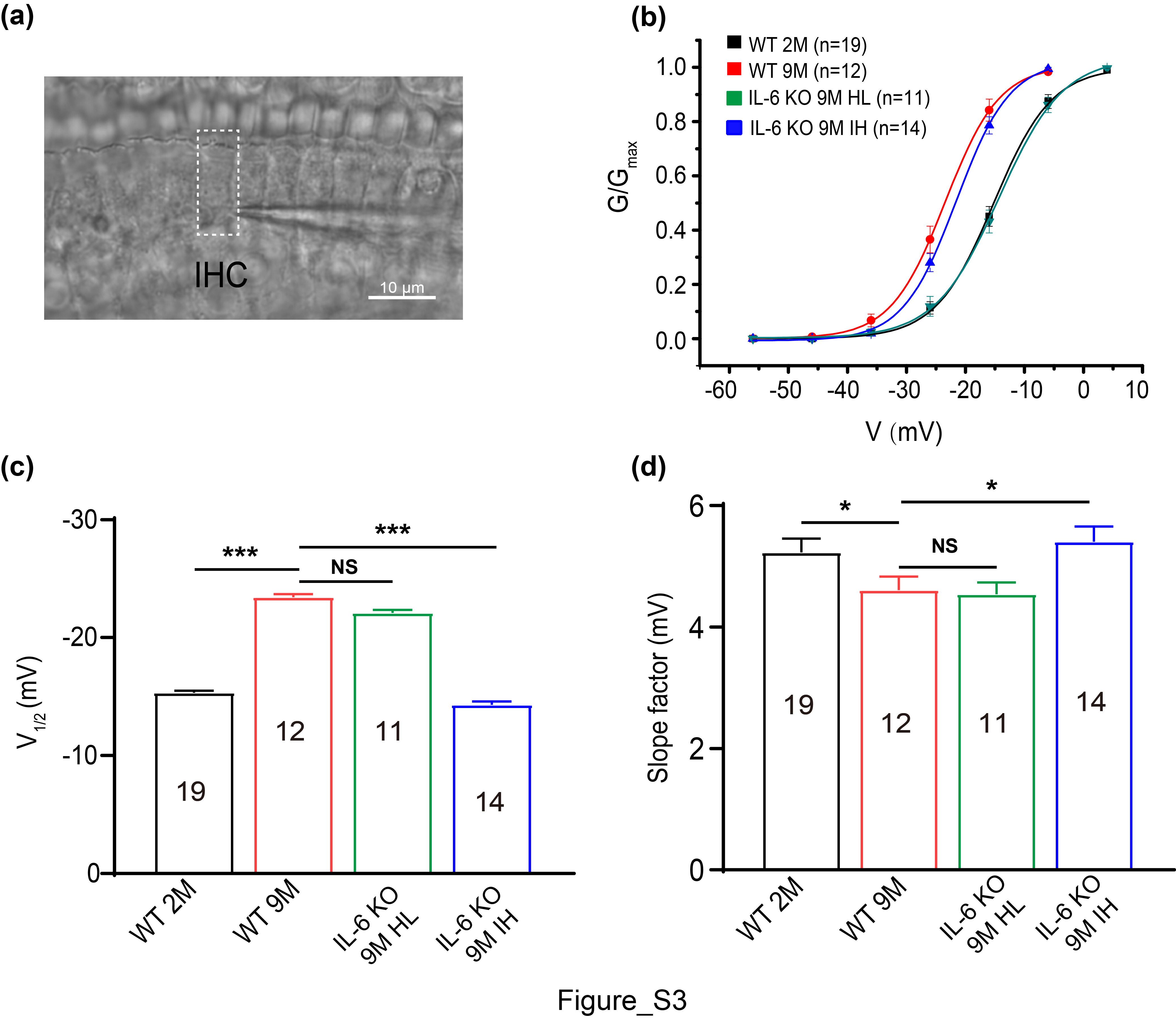

Supplement: Supplementary file 3 — Figure S3. IL‐6 induces upregulation of Cav1.3 channel of IHCs in AHL. (a) Photomicrograph showing a row of IHCs and a patch‐clamp recording electrode allowing measurements of ionic currents. (b) Activation curves of Ca2+ channels recorded in IHCs from WT and IL‐6 KO mice. (c, d) Summaries of V1/2 and slope factor of activation curve in IHCs from WT and IL‐6 KO mice. Data are means ± SEM, *p < 0.05, ***p < 0.001. [file ACEL-23-e14305-s006.jpg]

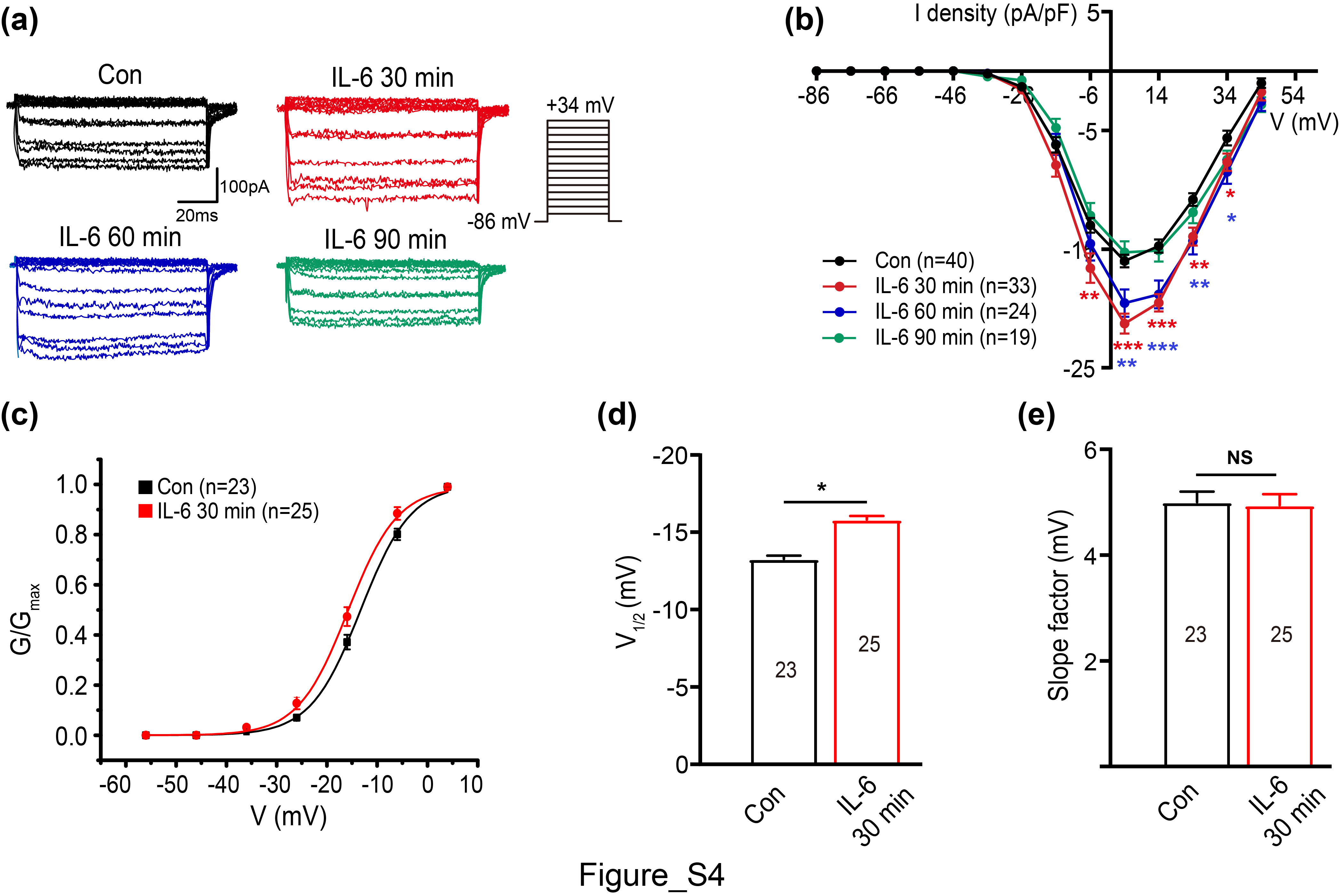

Supplement: Supplementary file 4 — Figure S4. IL‐6 incubation induces upregulation of Cav1.3 channel in IHCs in vitro. (a) Representative traces of Ca2+ currents in IHCs after incubation of IL‐6 (100 ng/mL) for 30 min, 60 min, and 90 min. (b) Current density‐voltage curves obtained from IHCs in the control and IL‐6 incubated groups. *30 min, 60 min versus Control. (c) Activation curves of Ca2+ channels fitted with the Boltzmann equation in IHCs. (d, e) V1/2 and slop factor of activation curve in IHCs of control and IL‐6 30 min groups. [file ACEL-23-e14305-s007.jpg]

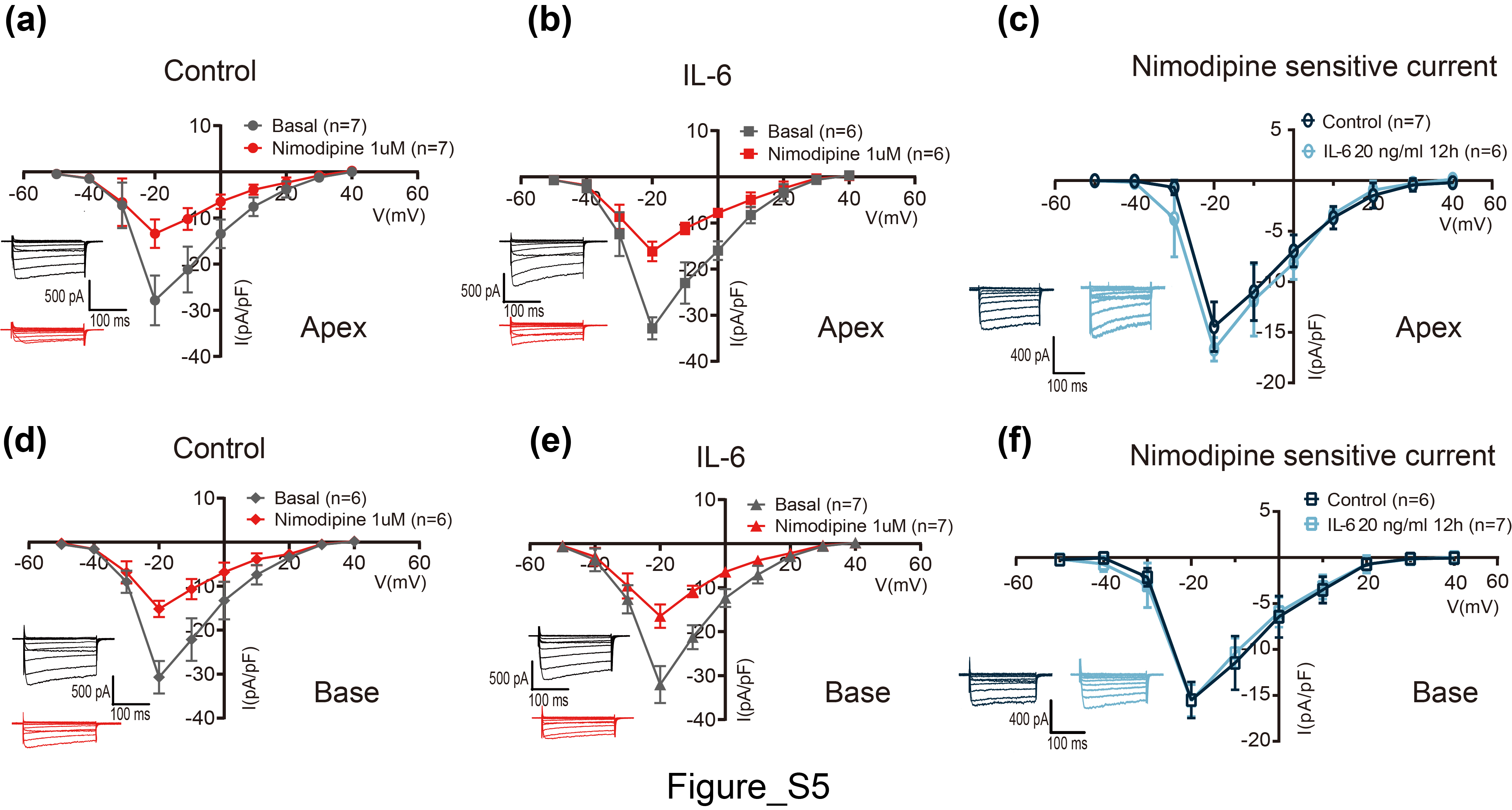

Supplement: Supplementary file 5 — Figure S5. IL‐6 incubation does not upregulate of nimodipine‐sensitive Ca2+ currents in SGNs in vitro. (a, b) Current density–voltage curves obtained from apical SGNs before and after application of nimodipine in control and IL‐6 treatment (incubated with IL‐6 at 20 ng/mL for 12 h). (c) The nimodipine‐sensitive Ca2+ currents in apical SGNs in control and IL‐6 treated groups. (d, e) Current density–voltage curves obtained from basal SGNs before and after application of nimodipine in control and IL‐6 treatment. (f) The nimodipine‐sensitive Ca2+ currents in basal SGNs in control and IL‐6 treated groups. [file ACEL-23-e14305-s005.jpg]
