# Supplementary material for: Upregulation of the Cav1.3 channel in inner hair cells by interleukin 6‐dependent inflammaging contributes to age‐related hearing loss
Source: Aging Cell. 2024 Aug 15;23(12):e14305. doi: 10.1111/acel.14305 (PMC11634703; doi:10.1111/acel.14305)
Supplement: Supplementary file 6 — Table S1. [file ACEL-23-e14305-s002.docx]

**Table S1**

The primers sequences for RT-PCR.

| Primer | Sequence (5′-3′) |
| --- | --- |
| IL-6-forward | CGGCCTTCCCTACTTCACAA |
| IL-6-reverse | TCTGCAAGTGCATCATCGTT |
| TNF-α-forward | TGTCCCTTTCACTCACTGGC |
| TNF-α-reverse | CTACTTTTGGGGGAGTGCCT |
| IL-1β-forward | TAACCTGCTGGTGTGTGAC |
| IL-1β-reverse | CATTGAGGTGGAGAGCTTTC |
| GAPDH-forward | TTGATGGCAACAATCTCCAC |
| GAPDH-reverse | CGTCCCGTAGACAAAATGGT |
