# Supplementary material for: Upregulation of the Cav1.3 channel in inner hair cells by interleukin 6‐dependent inflammaging contributes to age‐related hearing loss
Source: Aging Cell. 2024 Aug 15;23(12):e14305. doi: 10.1111/acel.14305 (PMC11634703; doi:10.1111/acel.14305)
Supplement: Supplementary file 7 — Table S2. [file ACEL-23-e14305-s001.docx]

**Table S2**

Immunofluorescence antibody

| Primary antibodies | Product Information |
| --- | --- |
| rabbit anti-Iba1 | #019-19741, Wako Chemicals,1:500 |
| rat anti-CD68 | #137001, Biolegend, 1:200 |
| mouse anti-IL-6 | #66146-1-lg, proteintech, 1:200 |
| mouse anti-Tuj1 | #801202, Biolegend, 1:100 |
| mouse anti-IL-6Rα | #sc-374259, Santa Cruz, 1:200 |
| rabbit anti-Tuj1 | #802001, Biolegend, 1:300 |
| rabbit anti-Myo7a | #PT-25-6790, Proteus, 1:200 |
| rabbit anti-MBP | #AB218011, Abcam, 1:500 |
| mouse anti-GluR2 | #MAB397, Millipore, 1:400 |
| mouse anti-CtBP2 | #612044, BD Biosciences, 1:400 |
| mouse anti-Myo7a | #138-1, Developmental Studies Hybridoma Bank, 1:20 |
| Secondary antibodies | Product Information |
| Alexa 488-FTTC | #115-545-003, Jackson Immuno Research, PA, 1:400 |
| Alexa 488-CY3 | #115-165-003, Jackson Immuno Research, PA, 1:400 |
| Alexa 568-FITC | #111-545-003, Jackson Immuno Research, PA, 1:400 |
| Alexa 568-CY3 | #111-165-003, Jackson Immuno Research, PA, 1:400 |
